# Reference Correlation for the Viscosity of Nitrogen from the Triple Point to 1000 K and Pressures up to 2200 MPa

**DOI:** 10.1007/s10765-024-03440-1

**Published:** 2024-10-10

**Authors:** Marcia L. Huber, Richard A. Perkins, Eric W. Lemmon

**Affiliations:** Applied Chemicals and Materials Division, National Institute of Standards and Technology, 325 Broadway, Boulder, CO 80305 USA

**Keywords:** Nitrogen, Transport properties, Viscosity

## Abstract

We present a new wide-ranging correlation for the viscosity of nitrogen based on critically evaluated experimental data as well as *ab-initio* calculations. The correlation is designed to be used with densities from an existing equation of state, which is valid from the triple point to 1000 K, at pressures up to 2200 MPa. The estimated uncertainty (at the 95% confidence level) for the viscosity varies depending on the temperature and pressure, from a low of 0.2% in the dilute-gas range near room temperature, to 4% for the liquid phase at pressures from saturation up to 34 MPa, and maximum of 8% in the supercritical region at pressures above 650 MPa. Extensive comparisons with experimental data are provided.

**Supplementary Information:**

The online version contains supplementary material available at 10.1007/s10765-024-03440-1.

## Introduction

Nitrogen is a major constituent of air and is used in many industrial processes; accurate representation of its viscosity is of technological importance. In addition, as a result of the passage of the CHIPS Act (Creating Helpful Incentives to Produce Semiconductors) of 2022 [1], there is increased interest in improving the calibration of the flow of gases used in semiconductor processing. Numerous gases are used in the production of semiconductors, and flow meters are often calibrated with one gas, such as nitrogen, and then calibration coefficients for other gases are determined using gas-property data [2]. Viscosity is one of the properties used in the development of some flow-meter models. It is the goal of this work to utilize recent measurements, as well as recent improvements in quantum-chemical ab initio computations that allow improvement in the representation of the dilute-gas viscosity, to develop a new wide-ranging correlation that can be used for calibration purposes.

In 1974, Hanley and coworkers [3] critically evaluated experimental data for the viscosity of nitrogen and proposed a wide-ranging correlation that was incorporated in the later work of Younglove [4]. Later, in 1987, Krauss et al. [5] also critically evaluated the experimental data and proposed a correlation that is valid over a wide range of fluid states. In 1996, Millat and Vesovic [6] presented a correlation. In 2004, Lemmon and Jacobsen [7] developed a viscosity correlation for nitrogen that is widely used as a standard and incorporated into computer programs such as REFPROP [8]. The dilute-gas properties of their formulation were developed using Chapman-Enskog theory with a collision integral fitted to experimental data available at the time, with agreement with atmospheric-pressure experimental data to generally within 0.5 %. Uncertainties of 1 % were given for temperatures between 270 and 300 K at pressures less than 100 MPa; they increased to about 2 % at temperatures of 180 K and higher. Below 180 K (excluding the critical region), the uncertainty increases to about 5 % as one nears the triple point. The uncertainty is larger in the critical region since the correlation did not include any special treatment for that region.

Since 2004, there have been several developments that can be used to improve the nitrogen correlation presently in use. Advances have occurred in *ab-initio* calculations that allow accurate calculation of the viscosity in the zero-density limit. For helium, the uncertainty in absolute viscosity of the dilute gas is almost two orders of magnitude smaller than existing experimental measurements [9]. Other gases have larger uncertainties than helium. For nitrogen, the authors did not explicitly give an uncertainty however they state the *ab-initio* results are about 0.3 % lower than the best experimental data between 300 and 700 K [10] that have uncertainties of 0.14 % to 0.19 %. Recently Xiao et al. [11–13], used a reference value for the viscosity of nitrogen determined from experimental viscosity ratios [14] combined with the ratio of two dilute-gas viscosities at the temperature of interest and at 298.15 K from the *ab-initio* calculations of Hellmann [10] to generate viscosities in the limit of zero density over a wide range of temperatures with very low uncertainty. Xiao et al. [11] also developed a reference correlation for the zero-density viscosity based on these results that can be incorporated into a new correlation. In addition, additional high-quality data have been published, including some very-high-pressure viscosity data sets that permit the development and validation of a correlation to higher pressures. Thus, it is the goal of this work to incorporate new data and theory to provide an improved correlation for the viscosity of nitrogen.

The first step in the development of a new correlation is the assembly and critical assessment of available literature data to determine the best data for use in correlation development. We identify two categories of experimental data: primary data, for use in the development of the correlation, and secondary data, used only for comparison purposes. According to the recommendation adopted by the Subcommittee on Transport Properties (now known as The International Association for Transport Properties) of the International Union of Pure and Applied Chemistry, the primary data are identified by a well-established set of criteria [15]. These criteria have been used successfully to establish standard reference values for the viscosity and thermal conductivity of fluids over wide ranges of conditions, with uncertainties in the range of 1 %. However, such a narrow definition greatly limits the range of the data representation. Consequently, within the primary data set, it is also necessary to include results that extend over a wide range of conditions, albeit with a higher uncertainty, provided they are consistent with other lower uncertainty data or with theory. In all cases, the uncertainty claimed for the final recommended data must reflect the estimated uncertainty in the primary information.

## The Correlation

The viscosity *η* can be expressed [16] as

1 $$\eta \,\left( {\rho ,{\rm T}} \right)\, = \,\left( {\eta_{0} {\kern 1pt} \left( {\rm T} \right)\, + \,{\Delta }\eta_{{{\text{res}}}} \left( {\rho ,{\rm T}} \right)} \right)\, \cdot \,{\Delta }\eta_{{\text{c}}} {\kern 1pt} \left( {\rho ,{\rm T}} \right),$$

where *ρ* is the density in mol·L^−1^, *T* is the absolute temperature in K, and the viscosity is in μPa·s. The first term, *η*_0_(*Τ*) = *η*(0,*Τ*), is the contribution to the viscosity in the dilute-gas limit, where only two-body molecular interactions occur. The second term Δ*η*_res_(*ρ*,*T*) is often called the residual term, and represents the contribution of all other effects to the viscosity of the fluid at elevated densities, including many-body collisions, molecular-velocity correlations, and collisional transfer. It can also be expressed as

2 $$\Delta \eta_{{{\text{res}}}} (\rho ,T) = \eta_{1} (T)\rho + \Delta \eta_{{{\text{high}}}} (\rho ,T),$$

where *η*_1_(*Τ*) *ρ* is a linear-in-density term, known as the initial density dependence term, and all higher- order contributions are contained in the term Δ*η*_high_(*ρ,Τ*). The initial density dependence term can be separately determined with the Rainwater-Friend theory [17–19] for the transport properties of moderately dense gases or can be found by fitting the full term Δ*η*_res_ to experimental data [20]. The last term, Δ*η*_c_(*ρ,Τ*), is a multiplicative term that accounts for the critical enhancement. This is due to long-range density fluctuations that occur in a fluid near its critical point, which contribute to divergence of the viscosity at the critical point. The critical enhancement term for viscosity is significant only in the region very near the critical point, as shown in Vesovic et al. [21] and Hendl et al. [22]. If data in the critical region are not available, this term is set to one.

In addition to performing literature searches and utilizing content in previous correlations, we made extensive use of the NIST ThermoData Engine [23] to identify data sources. Table 1 summarizes, to the best of our knowledge, all the available experimental measurements of the viscosity of nitrogen reported in the literature. Table 1 also provides the experimental method, the sample purity, uncertainty as reported by the original authors, the number of measurements, as well as the range of temperatures, pressures, and densities. The uncertainty in Table 1 is at the 95 % (*k* = 2) level; however, many authors often failed to report *k* and, in such cases, we assumed *k* = 2. The uncertainties are as reported by the authors, and at times may be overly optimistic.

**Table 1:** Viscosity measurements of nitrogen

First author	Publ year	Technique^a^	Purity (%)	Uncertainty (%)	No. data	Temperature range (K)	Pressure range (MPa)	Density range (mol L^−1^)
Primary data								
Zhou [27]	2024	VW	99.999	4.2	20	89.3–199.9	0.35–4.98	0.65–27.3
Cheng^b^ [28]	2020	Cap	99.999	5	52	279.8–923.1	0.9–31.0	0.38–5.26
Humberg [29]	2018	Rot.Body	99.9999	0.14–0.19	56	253–473	0.1–1.8	0.03–0.67
Sakoda [30]	2015	VW	99.99	1.4	20	296–573	0.1–0.7	0.02–0.3
Abramson [31]	2014	RB	na	5	17	294.7–676.8	1010–10700	37.9–63.1
Vogel^c^ [32–35]	2012	OD	99.9995	0.15–0.2	96	292–689	0.03–0.22	0.01–0.04
Yusibani [36]	2011	Cap	na	2	32	297–499	0.6–95.2	0.2–20.0
Seibt^c^ [37]	2009	VW	99.999	0.25–0.3	66	293–423	0.1–30.3	0.03–10.9
Abramson [38]	2008	RB	99.995	na	61	293.6–573.5	340–6950	30.1–57.5
May [39]	2007	Cap	99.999	0.09	2	298	0–0.1	Dilute gas
Seibt^c^ [40]	2006	VW	99.999	0.2–0.3	321	298–423	0.1–35	0.02–11.8
Hurly [41]	2003	Grsp	99.99	0.5	23	298.15	1.7–3.2	0.7–1.3
Evers [42]	2002	RotCyl	99.9995	0.15–0.4	76	233–523	0.09–29.7	0.03–10.7
Hoogland [43]	1985	Cap	na	0.1	15	298–333	0.2–11.7	0.07–4.7
Rutherford [44]	1984	Cap	na	1	15	298	0.4–7.0	0.2–2.8
Diller [45]	1983	TorCr	Res.grade	2	65	90–300	0.36–33.6	0.68–29.6
Matthews^d^ [46]	1982	Cap	99.99	1	11	119–1597	0.1	Dilute gas
Lavushchev [47]	1978	Cap	na	1	54	375–1990	0.1	Dilute gas
Hongo [48]	1977	OD	99.99	0.3	78	298–373	0.1–12.4	0.04–4.8
Kobayashi [49]	1977	Cap	99.999	0.05–0.2	6	298	0.11–4.0	0.04–1.6
Gough [50]	1976	Cap	99.8	1	11	120–320	0.1	Dilute gas
Timrot [51]	1975	OD	99.99	1.5	31	295–573	0.1–11.8	0.02–4.6
Carey [52]	1974	AcRes	na	0.1	44	289.1–992.3	0.1–52.8	0.01–10.4
Zozula^c,e^ [53]	1974	OD	99.97	2	79†	127–135	3.4–6.4	6.9–16.1
Kestin [54]	1971	OD	na	0.2	33	298	0.1–10.7	0.04–4.28
Clarke [55]	1969	Cap	99.7	0.5	13	120–360	0.1	Dilute gas
Gracki [56]	1969	Cap	99.998	0.2	46	183–298	0.53–25.7	0.3–12.8
Chierici [57]	1969	Cap	99.99	1	12	323	0.6–91.3	0.22–18.76
Guevara [58]	1969	Cap	na	0.4	22	1100–2149	0.1	Dilute gas
Vermesse [59]	1969	Cap	na	2	89	273–370	10.7–651	3.4–35.3
Kestin [60]	1968	OD	99.999	0.05^f^	6	303	0.1–2.4	0.04–0.9
Kao [61]	1967	Cap	99.997	0.14	35	183–323	1.0–50.7	0.4–17.3
Van Itterbeek [62]	1966	OD	na	na	38	70–90	0.1–9.9	26.6–30.6
Flynn [63]	1963	Cap	99.998	0.1	34	195–373	0.68–17.9	0.27–12.8
Iwasaki [64]	1963	OD	99.998	0.2	32	293–298	0.1–10.0	0.04–4.0
Van Itterbeek [65]	1962	OD	na	1	13	64–77	0.1	28.8–30.9
Kestin [66]	1959	OD	99.999	0.05	20	293–298	0.01–7	0.0–2.8
Kestin [67]	1959	OD	99.999	0.1	14	293–296	0.1–15.5	0.04–6.2
Kestin [68]	1958	OD	99.9	0.2	10	298	0.1–7.1	0.04–2.9
Lazarre [69]	1957	Cap	99.99	1	21	298–348	0.09–319	9.03–29.5
Rudenko [70]	1934	Cap	na	1.4	8	63.9–77.3	0.01–0.1	28.8–30.8
Michels [71]	1931	Cap	99.99	0.15–0.5	56	273–348	1.1–97.9	0.45–20.3
Secondary data								
Pinho [72]	2015	Cap	99.8	2	1	305	9	3.6
Lv [73]	2014	OD	99.99	1	24	303	0.1–4.5	0.04–1.9
Wang [74]	2014	OD	99.999	2	24	303	0.1–4.7	0.04–1.87
El Hawary [75]	2009	RotCyl	99.999	0.1–0.25	116	253–473	0.1–19.7	0.04–7.7
Tomida [76]	2009	OD	99.99	0.5	30	298–423	0.1–5.0	0.02–1.6
Sih [77]	2008	FBody	99.999	5	20	298–313	0.5–7.7	0.2–2.9
Audonnet [78]	2001	VW	99.95	2.5	8	303–323	0.17–21.25	1.8–7.6
Assael [79]	1997	VW	99.9	1	7	313–454	0.1	Dilute gas
Docter [80]	1997	RotCyl	99.9993	0.4	31	253–523	0.1–28.5	0.02–5.7
Dunlop [81]	1994	Cap	99.99	0.3	1	298	0.1	Dilute gas
Hansen [82]	1994	Cap	na	2	6	313–473	0.02	Dilute gas
Strehlow [83]	1987	OD	99.99	0.1–0.2	47	294–689	0.1	Dilute gas
Lukin [84]	1983	Cap	na	0.3	23	76.5–293	0.09–0.1	Dilute gas
Kestin [85]	1982	OD	99.999	0.1–0.2	5	298–473	0.1	Dilute gas
Abe [86]	1979	OD	99.9	0.3	5	298–468	0.1	Dilute gas
Kestin [87]	1977	OD	99.999	0.1–0.2	9	298–673	0.1	Dilute gas
Kestin [88]	1976	OD	99.99	0.1–0.3	9	298–1270	0.1	Dilute Gas
Matthews [89]	1976	Cap	99.9	0.5–1.5	15	120–1700	0.1	Dilute gas
Schlumpf [90]	1975	Cap	na	3.5	11	323	10–300	3.7–28.3
Golubev [91]	1974	Cap	99.997	2	70	273–423	9.8–401	2.7–30.5
Maitland [92]	1974	Cap	99.9	1–1.5	24	394–1550	0.1	Dilute gas
Borisov [93]	1973	Cap	na	na	1	300	0.1	Dilute gas
Hellemans [94]	1973	OD	99.99	0.1–0.3	6	298–770	0.1	Dilute gas
Kestin [95]	1972	OD	99.999	0.1	8	298–973	0.1	Dilute gas
Kestin [96]	1972	OD	99.999	0.1–0.3	6	298–973	0.1	Dilute Gas
Golubev [97]	1971	Cap	99.997	1	4	296–573	0.1	Dilute gas
Dawe [98]	1970	Cap	99.9	0.5	15	293–1600	0.1	Dilute gas
Grevendonk [99]	1970	TorCry	na	3	134	66.5–123	0.6–19.5	17.9–31.3
Hellemans [100]	1970	OD	na	na	44	96.7–125	0.6–9.8	15.8–26.7
Hellemans [101]	1970	OD	na	3	18	64.8–123	0.01–2.9	17.1–30.7
Munczak [102]	1969	Cap	99.95	1	3	288–323	0.1	Dilute gas
Timrot [103]	1969	OD	99.99	0.8	8	300–650	0.1	Dilute gas
Clarke [104]	1968	Cap	99.7	0.5	12	114–375	0.1	Dilute gas
DiPippo [105]	1968	OD	na	0.1	41	294.9–773.7	0.03–0.18	Dilute gas
Shepeleva [106]	1968	Cap	99.99	1	64	80.5–278	0.9–50.6	0.4–31.5
Boon [107]	1967	Cap	na	na	4	68.1–70.2	0.8	30–30.3
DiPippo [108]	1967	OD	99.999	0.1	5	303	0.1–2.3	0.04–0.9
Gururaja [109]	1967	OD	na	1	2	298	0.1	Dilute gas
DiPippo [110]	1966	OD	99.9985	0.1	24	296–773	0.03–0.17	Dilute Gas
Reynes [111]	1966	Cap	99.996	na	30	373–473	7.1–69.4	1.8–14.8
Rigby [112]	1966	Cap	99.6	0.3	15	293–973	0.1	Dilute gas
Van Itterbeek [62]	1966	OD	na	na	33	70–90	0.1–2.4	26.6–30.1
Forster [113]	1963	OD	99.8	2	10	66–121	0.01–2.65	18.1–30.5
Goldman [114]	1963	Cap	na	na	16	195–298	5.2–12.7	3.2–9.6
Kestin [115]	1963	OD	99.999	0.5	37	344–539	0.1–14.8	0.02–4.6
Makavetskas [116]	1963	Cap	na	3.5	62	285–933	1.5–60	0.2–16.7
Vermesse [117]	1963	Cap	na	na	24	299–322	54.9–488	15.5–32.3
Filippova [118]	1962	Cap	99.5	3	27	90.2–273	3.5–15.1	3.58–28.1
Baron [119]	1959	Cap	99.9	1‡	40	325–408	0.7–55.2	0.2–14.5
Ellis [120]	1959	Cap	99.5	1	7	973–1273	0.1	Dilute gas
Glaser [121]	1959	FB	na	na	124	287–453	0.1–34.3	0.02–11.5
Makita [122]	1957	RB	99.8	na	54	299–533	0.1–78.5	0.02–18.5
Ross [123]	1957	Cap	99.7	1	41	223–298	3.4–68.9	1.4–21.3
Kiyama [124]	1956	RB	na	1.5	24	298–348	9.8–78.5	3.3–18.5
Iwasaki [125]	1954	OD	99.6	1	25	298–423	02.1–19	0.6–7.2
Golubev [126]	1953	Cap	na	na	27	93–1074	0.1	Dilute gas
Bonilla [127]	1951	Cap	na	na	25	200–2500	0.1	Dilute gas
Buddenberg [128]	1951	Cap	99.9	na	6	290–300	0.1	Dilute gas
Schmid [129]	1942	Cap	na	na	11	273–1274	0.1	Dilute gas
Wobser [130]	1941	FBall	na	na	5	293–371	0.1	Dilute gas
Gerf [131]	1940	Cap	99.8	2.5	7	66–77	0.02–0.1	28.8–30.5
Johnston [132]	1940	OD	na	0.3–0.8	16	90–300	0.1	Dilute gas
Rudenko [133]	1939	ConCyl	na	na	7	77.4–112	0.1–1.62	21.7–28.8
Herning [134]	1936	Cap	99.8	na	1	293	0.1	Dilute gas
Trautz [135]	1931	Cap	na	na	9	293–524	0.1	Dilute gas
Boyd [136]	1930	Cap	na	na	68	303–343	7.13–19.4	2.5–6.9
Trautz [137]	1930	Cap	na	na	4	301–550	0.1	Dilute gas
Trautz [138]	1930	Cap	na	na	12	293–1099	0.1	Dilute gas
Smith [139]	1922	Cap	na	na	2	288–373	0.1	Dilute gas
Yen [140]	1919	Cap	na	0.15	1	296	0.1	Dilute gas
Vogel [141]	1914	OD	na	na	2	82–273	0.1	Dilute Gas
Schmitt [142]	1909	Cap	na	na	6	287–456	0.1	Dilute gas
Markowski [143]	1904	Cap	na	na	30	287–456	0.1	Dilute gas

^a^*AcRes* Acoustic resonator, *Cap* Capillary, *ConCyl* Concentric cylinder, *Fball* Falling ball, *Fbody* Falling body, *Grsp* Greenspan Viscometer, *OD* Oscillating Disk, *RB* Rolling ball, *Rot. Body* Rotating body, *Rot.Cyl*. Rotating cylinder, *TorCry* Torsional crustal, *VW* Vibrating wire.

^b^Only points above 600 K included in primary data ^c^ used experimentally measured densities ^d^ point at 1597 K excluded ^e^ critical region data in Table 3 of Ref. [53] excluded Note that ref [33–35] do not appear in secondary as they have been replaced by ref. [32] ^f^ precision

The correlation in Eq. 1 is formulated in terms of temperature and density. Most experimental measurements of viscosity are performed at a known temperature *T* and pressure *P*, and it is necessary to obtain the density associated with the *T*, *P* state point. We use the Helmholtz equation of state (EOS) formulated by Span et al. [24] to obtain density after first converting all temperatures to ITS-90 [25, 26] if necessary. We also use the critical point associated with this EOS; the critical point and other constants for this EOS are given in Table 2. This EOS reports an uncertainty in density of 0.02 % from the triple point up to temperatures of 523 K and pressures up to 12 MPa and from temperatures of 240 K to 523 K at pressures less than 30 MPa. In the range from 270 to 350 K at pressures less than 12 MPa, the uncertainty in density is 0.01 %. The uncertainty in density at very high pressures (> 1 GPa) is 0.6 %. Additional details about uncertainties in the EOS can be found in [24].

**Table 2:** Critical point and fixed constants for the EOS of Span et al. [24]

Property	Symbol	Units	Value
Critical temperature	*T*_c_	K	126.192
Critical pressure	*P*_c_	MPa	3.3958
Critical density	*ρ*_c_	mol·dm^−3^	11.1839
Triple-point temperature	*T*_tp_	K	63.151
Molar mass	*M*	g·mol^−1^	28.01348

Since the publication of the viscosity model in 2004 [7], there have been a number of new measurements, as shown in Table 1. Overall there are a fairly large number of measurements; for a discussion of some of the older data, one can consult Hanley and coworkers [3] and Krauss et al. [5]. Inspection of the deviation plots in Lemmon and Jacobsen [7] is also helpful to discern consistency between data sets. Here we will focus on the more recent data. Most recently Zhou et al. [27] measured nitrogen in a vibrating-wire apparatus and although the uncertainty is somewhat large, we have included their liquid-phase measurements in primary since low-temperature and especially liquid-phase data are relatively sparse. Similarly Cheng et al. [28] made measurements in a capillary viscometer with relatively large uncertainties, but they include a region (high temperature, high pressure) where measurements are sparse. We included only the measurements above 600 K in the primary set. In 2018, Humberg et al. [29] utilized a rotating-body viscometer to obtain very accurate measurements over a wide range of temperatures (253 K to 473 K) at pressures up to 1.8 MPa; they also analyzed their data to provide values for the viscosity in the limit of zero density and the second viscosity virial coefficient; these are an important addition to the primary data set. Additional measurements added to the primary set are Sakoda et al. [30] who used a vibrating-wire apparatus for measurements covering 296 K to 573 K at pressures up to 0.7 MPa. Two very high-pressure sets from Abramson [31, 38] made with a rolling-sphere method in a diamond-anvil cell, achieving pressures of 7 GPa [38] and 10.7 GPa [31], were included to extend the pressure range of the correlation to 10.7 GPa. This is well above the recommended upper pressure limit (2.2 GPa) of the EOS; however, the EOS was designed to extrapolate well to higher pressures. In 2012, Vogel [36] published new very high-precision measurements for the viscosity of nitrogen at low density (0.72 kg·m^−3^) in an all-quartz oscillating-disk viscometer. In this same work, Vogel recalibrated his earlier measurements from 1972 [37], 1984 [38], and 1989 [53], resulting in more accurate values than the original publications. Yusibani et al. [36] performed measurements in a capillary viscometer that covered pressures up to 95 MPa; these were included to increase data coverage at high pressures. Two high-precision data sets from Seibt et al. [37, 40] from vibrating wire instruments are included in the primary data, as well as the reference data of May et al. [39]. All other data sets obtained after 2004 were classified as secondary. For example, Pinho et al. [72], El Hawary [75], and Sih et al. [77] were also added to secondary as there are other sets with lower uncertainties covering their range of *T*,*P*. In 2014, both Wang et al. [74] and Lv et al. [73] measured the viscosity of nitrogen with an oscillating-disk viscometer at 303 K and pressures to 4.5 MPa with reported uncertainties of 2 % and 1 % respectively. Since there are other data sets in this region that have lower uncertainties, these sets were classified as secondary. Finally, we assigned the measurements of Tomida et al. [76] to secondary as they exhibited a slight offset from other data in the same range of *T* and *P*.

**Fig. 1:**
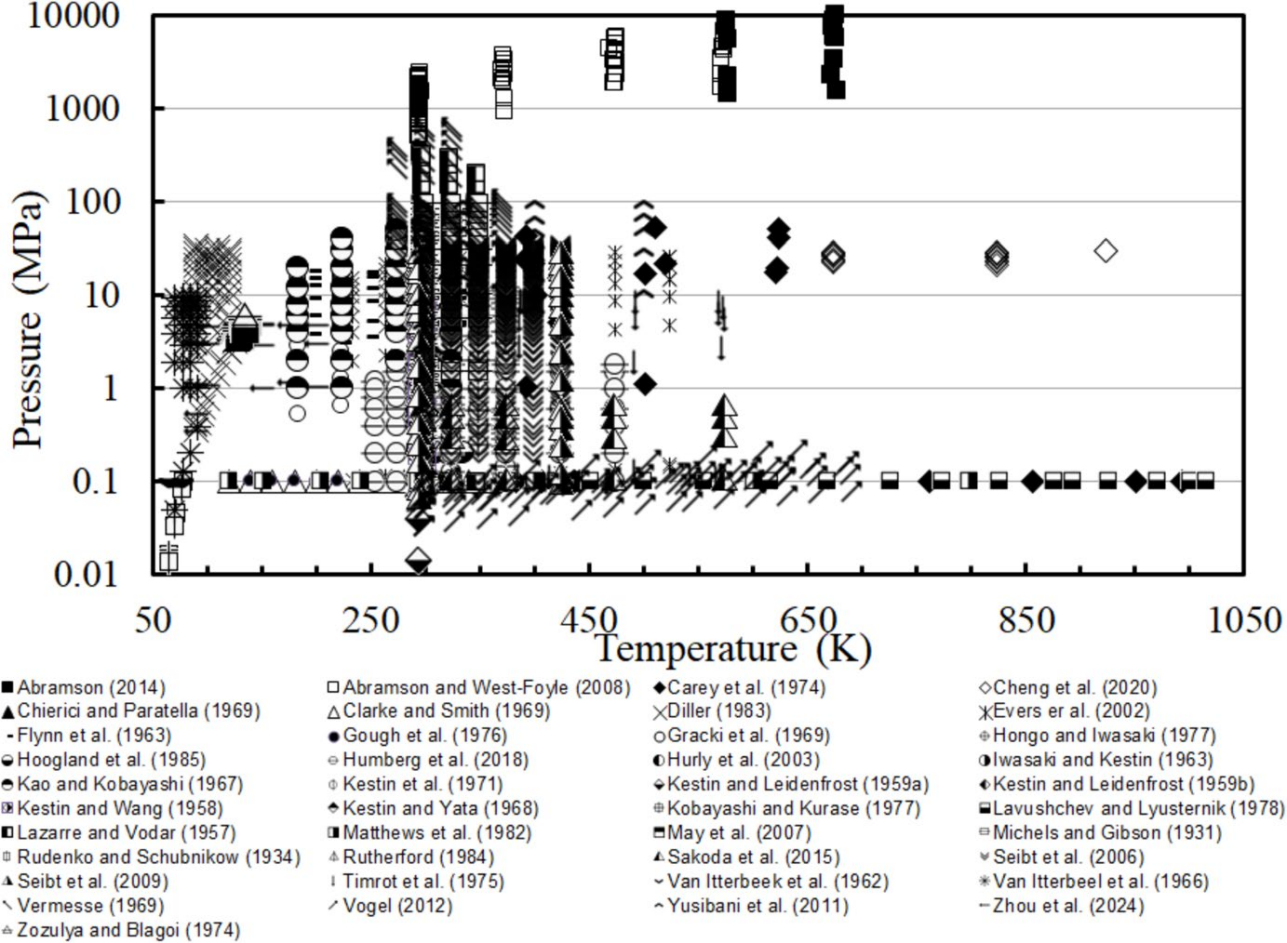
Temperature–pressure ranges of the primary experimental viscosity data for nitrogen

**Fig. 2:**
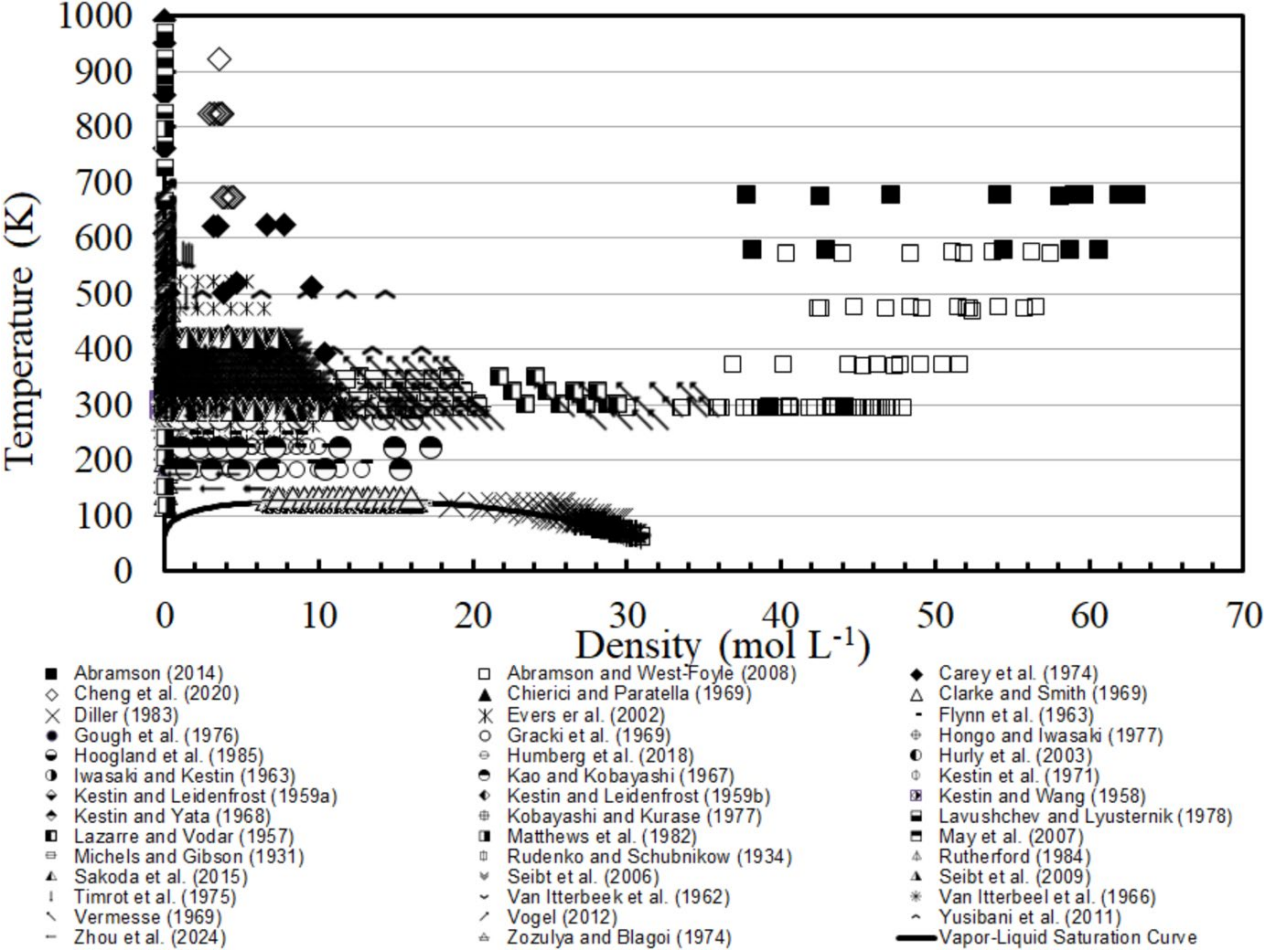
Temperature-density ranges of the primary experimental viscosity data for nitrogen. Solid line denotes the vapor–liquid saturation boundary

Vogel [32] presented a very careful analysis of viscosity measurements of nitrogen at low density that we incorporated into our assessment of primary data. Data at both low and high temperatures differ from each other by more than their claimed uncertainties and it is difficult to assess which sets display correct behavior. Based on comparisons with the theoretical temperature behavior of *ab-initio* calculations [10], Vogel [32] and also Hellman [10] both concluded that it is now possible to make recommendations on data sets at both very low and high temperatures where there is disagreement between the available data. For example, in the temperature range 1100 K to 2000 K the data of Guevara et al. [58] and Lavushchev and Lyusternik [47] are more consistent with theoretical behavior than the results of the Smith group [46, 92, 98]. At low temperatures Vogel concluded that the data of Johnston and McCloskey [132] and Lukin et al. [84] are not consistent with the theoretical behavior or with other low-temperature experiments [46, 50, 55, 56, 61, 104], and should not be included in future correlation development. We have adopted these recommendations in our selection of primary data. Since theoretical guidance was unavailable at the time of the Lemmon and Jacobsen [7] correlation, when data sets were inconsistent it was not possible to ascertain which data had correct behavior and all data were used in fitting. The *P*,*T* and *T*,*ρ* ranges of the resulting primary data are shown in Figs. 1 and 2. The data of Guevera et al. [58] that extend from 1100 to 2149 K and data in Lavushchev and Lyusternik [47] that exceed 1050 K are not shown. Finally, note that two data sets [31, 38] contain points that exceed the recommended upper pressure limit of the EOS of Span et al. [24] (2200 MPa) and the EOS was used in an extrapolation mode to obtain densities for these points.

### The Dilute-Gas Limit Viscosity Term

The behavior of nitrogen in the dilute-gas region was discussed in several older publications, including Cole and Williams [144], Boushehri et al.[145], and the correlation of Stephan et al. [5]. As mentioned earlier, Vogel [32] analyzed the low-density limit and showed comparisons with the Lemmon and Jacobsen correlation [7] and suggested that an improved dilute-gas correlation could be developed with the aid of *ab-initio* calculations. The *ab-initio* calculations of Cencek et al. [9] provide an incredibly accurate value for the viscosity of helium at 25 °C and zero density, 19.8253 ± 0.0002 µPa·s (*k* = 1), that is much more accurate than can be determined experimentally. For example, accurate measurements of helium (see Ref. [146]) have uncertainties on the order of 0.1 %. Viscosity ratios can be measured much more accurately than absolute viscosity [14]; this was utilized by Berg and Moldover [14], who critically reviewed viscosity measurements and determined viscosity ratios. They then anchored them to the very accurate *ab-initio* value of helium at 25 °C and zero density [9] in order to provide a recommended reference value of the viscosity of nitrogen at 25 °C and zero density of 17.7494 ± 0.0048 µPa·s (*k* = 1). In 2020, Xiao et al. [11–13] extended this technique to provide a reference correlation for the viscosity of nitrogen valid over a wide temperature range (70 to 3000 K), which has an estimated uncertainty of 0.4 % (*k* = 2) over the entire temperature range, but has lower uncertainty in specific sub-regions based on comparisons with experimental data that will be discussed in a later section.

The dilute-gas limit viscosity, *η*_0,_ given by Xiao et al. [11–13], which we adopt here is,

3 $$\eta_{0} (T) = \eta_{0} (298.15\,{\text{ K}})\;\;\exp \left\{ {\sum\limits_{i = 1}^{10} {a_{i} \left( {\ln \left[ {\frac{T}{{298.15\;{\text{K}}}}} \right]} \right)}^{i} } \right\}.$$

The viscosity at 298.15 K, *η*_0_(298.15 Κ), was set to the recommended value proposed by Berg and Moldover [14], 17.7494 μPa·s. The coefficients *a*_*i*_ are shown in Table 3. Note that Xiao et al. [11–13] state that a possible limitation to this functional form is that it does not extrapolate to very low temperatures in a manner as expected by kinetic theory, and they provide an alternative formulation that can be used if one is concerned about the theoretical behavior in extrapolations to 0 K. The alternative formulation has slightly lower precision than Eq. 3, and we have opted to use Eq. 3 as recommended by Xiao et al. [11–13]. Xiao et al. [11–13] give the range of validity of this equation as 70 K < *T* < 3000 K with an uncertainty (*k* = 1) of 0.2 %.

**Table 3:** Coefficients *a*_*i*_ of Eq. 3 [11–13]

*i*	*a*_*i*_
1	7.734 578 × 10^–1^
2	− 9.310 761 × 10^–2^
3	2.716 958 × 10^–2^
4	6.175 553 × 10^–3^
5	− 7.201 594 × 10^–3^
6	2.094 372 × 10^–3^
7	1.922 676 × 10^–4^
8	− 3.454 323 × 10^–4^
9	1.051 771 × 10^–4^
10	− 1.126 739 × 10^–5^

### The Residual Contribution

Although one can treat the linear-in-density term separately using Rainwater-Friend theory [17–19], here we fit the entire residual term to a totally empirical form as was done in the Lemmon-Jacobsen correlation [7]

4 $$\Delta \eta_{{{\text{res}}}} (T_{{\text{r}}} ,\rho_{{\text{r}}} ) = \sum\limits_{i = 1}^{n} {N_{i} T_{{\text{r}}}^{{t_{i} }} } \rho_{{\text{r}}}^{{d_{i} }} \exp ( - \gamma_{i} \rho_{{\text{r}}}^{{l_{i} }} ),$$

where *T*_r_ is the reduced temperature *T*/*T*_c_, *ρ*_r_ is the reduced density *ρ*/*ρ*_c,_ and *N*_*i*_, *t*_*i*_ and *d*_*i*_ are parameters determined by fitting experimental data. We fit only the primary data using a modification of an in-house software package developed for multi-property nonlinear fitting of equations of state. Non-integer exponents on density were allowed above two, and positive temperature exponents were not permitted. Weights were adjusted manually to represent the data sets to near or within their estimated uncertainties.

Additionally, constraints were added to control the behavior of the correlation when extrapolated to very high pressures, and when the *T*, *P* state point is in the 2-phase region. Although the 2-phase region is not physically meaningful for viscosity, in some applications such as mixture models or some corresponding-states methods, it may be necessary to evaluate the correlation in the 2-phase region, or at values below the triple point. For this reason, we wanted to control the behavior such that viscosity is not negative, there are no mathematical poles, or wild swings in the value of viscosity in the 2-phase region. We forced the curvature of the slope of the logarithm of the viscosity vs. density at low temperatures in the 2-phase region to be positive above 1.3 mol·L^−1^. We also forced the value of the viscosity to be positive at very low temperatures (30 K) and extremely high densities up to 100 mol·L^−1^. A final constraint was added to control the behavior of the slope of viscosity vs. density at very low density. According to Rainwater-Friend theory [18], the value of the second viscosity virial coefficient is negative at low temperatures. Since there are no accurate low-density gas points at low temperatures, we used a constraint that forced the second viscosity virial coefficient to be negative at temperatures below 90 K. For guidance, we used Rainwater-Friend theory using the generalized correlation of Vogel et al. [147] with the parameters *σ* = 0.368 nm and *ε*/*k*_B_ = 90.9 K as recommended by Bich and Vogel [19]; this correlation shows the second viscosity virial coefficient going negative at temperatures less than about 113 K, so as a rough estimate we required the second viscosity virial to be negative below 90 K. Although we initially included exponential terms in Eq. 4, our final correlation contained only polynomials and the final expression is

5 $$\Delta \eta_{{{\text{res}}}} (T_{{\text{r}}} ,\rho_{{\text{r}}} ) = \sum\limits_{i = 1}^{10} {N_{i} T_{{\text{r}}}^{{t_{i} }} } \rho_{{\text{r}}}^{{d_{i} }} ,$$

with the coefficients given in Table 4, and Δ*η*_res_ expressed in μPa·s.

**Table 4:** Coefficients *N*_*i*_, *t*_*i*_ and *d*_*i*_ of Eqs. 1,3,5

*i*	*N*_*i*_	*t*_*i*_	*d*_*i*_
1	9.955235691668	− 0.77512218631	1
2	− 6.165266404871	− 2.00109608805	1
3	0.213120936996	− 5.81445500000	2
4	− 8.473713006806	− 0.67602596996	2
5	10.013103356639	0	2
6	0.638966874603	− 0.71613185456	6.96423363249
7	0.311620258213	− 1.14069399597	8.38651257501
8	9.241856768911	− 2.36525835980	2.99224857471
9	− 5.252828814854	− 2.54636992550	3.42744617975
10	− 0.667072279228	− 1.00794034515	7.97371767411

### Comparison with Data

Table 5 presents comparisons of the primary data with the present correlation (Eqs. 1, 3, 5) and with the correlation of Lemmon and Jacobsen [7]. The critical enhancement term Δ*η*_c_ is set to one for these comparisons, since the enhancement may be ignored except for the region very close to the critical point, defined as within 3% of the critical temperature and 25 % of the critical density [148, 149]. Comparisons with data in the critical region are given in Sect. 2.4.5. We use the following definitions:

6 $${\text{PCT}} = 100(\eta_{\exp } - \eta_{{{\text{calc}}}} )/\eta_{\exp }$$

7 $${\text{AAD = (}}\sum {\left| {{\text{PCT}}} \right|} )/{\text{NPTS}}$$

8 $${\text{BIAS = (}}\sum {{\text{PCT}}} )/{\text{NPTS}}$$

9 $${\text{RMS = }}\sqrt {{(}\sum {{\text{PCT}}}^{2} )/{\text{NPTS}}}$$

10 $${\text{STDEV = }}\sqrt {(\sum {({\text{PCT}} - {\text{BIAS}})^{2} } )/({\text{NPTS}} - 1)}$$

where *η*_exp_ is the experimental value of the viscosity, *η*_fit_ is the value calculated from the correlation, and NPTS is the number of points. One point (at 624 K and 50.9 MPa) from Carey et al. [52] was removed from the statistics and considered an outlier due to a probable typographical error in the source document since it deviated more than 3 standard deviations from the rest of the data set. In addition, we included the full data sets of Abramson [31] and Abramson and West-Foyle [38] even though they contain points above the upper pressure limit of the EOS of Span et al. [24] that was used to determine density. The results will be discussed according to five regions: low-pressure gas (*P* < 1 MPa), liquid, high pressure (*P* > 100 MPa), moderate pressure (1 MPa < *P* < 100 MPa), and the critical region.

**Table 5:** Evaluation of the nitrogen viscosity correlation Eqs.1,3,5 for the primary data

First author	NPTS	Present model					Lemmon and Jacobsen (2004) model				
		AAD	BIAS	STDEV	RMS	MAX	AAD	BIAS	STDEV	RMS	MAX
Zhou [27]	20	0.8	0.42	1	1.1	2.7	0.77	− 0.22	0.91	0.92	− 2.2
Cheng^a^ [28]	20	1.8	1.8	0.25	1.8	2.2	1.1	1.1	0.28	1.1	1.5
Humberg [29]	56	0.063	− 0.059	0.041	0.072	− 0.15	0.32	− 0.32	0.14	0.35	− 0.62
Sakoda [30]	20	0.38	0.35	0.24	0.42	0.7	0.18	0.0025	0.21	0.21	− 0.4
Abramson [31]	17	3.5	0.37	4.3	4.2	− 9.4	16	− 1	19	18	33
Vogel^b^ [32–35]	96	0.055	0.0062	0.069	0.069	− 0.22	0.41	− 0.41	0.16	0.44	− 0.75
Yusibani [36]	32	1.3	0.1	1.6	1.6	3.9	1.3	0.18	1.6	1.6	3.9
Seibt^b^ [37]	66	0.32	− 0.19	0.31	0.36	0.66	0.44	− 0.39	0.33	0.51	− 0.96
Abramson [38]	61	3.5	− 1.4	3.9	4.1	− 9.8	11	− 1.5	13	13	− 30
May [39]	2	0.0047	0.0047	0.0018	0.0049	0.006	0.23	− 0.23	0.0083	0.23	− 0.23
Seibt^b^ [40]	321	0.14	− 0.1	0.13	0.17	− 0.4	0.29	− 0.23	0.25	0.34	− 0.73
Hurly [41]	23	0.17	− 0.17	0.086	0.19	− 0.28	0.28	− 0.28	0.041	0.28	− 0.34
Evers [42]	76	0.28	0.14	0.33	0.35	0.73	0.085	− 0.0027	0.11	0.11	0.37
Hoogland [43]	15	0.1	0.071	0.093	0.11	0.18	0.12	− 0.021	0.14	0.13	− 0.21
Rutherford [44]	15	0.23	0.04	0.3	0.29	− 0.66	0.17	− 0.031	0.25	0.24	− 0.6
Diller [45]	65	1.3	0.38	1.5	1.6	3.5	1.2	− 0.13	1.5	1.5	− 3.8
Matthews^c^ [46]	10	0.38	0.35	0.24	0.42	0.74	0.41	− 0.23	0.69	0.7	− 1.9
Lavushchev [47]	54	0.28	− 0.011	0.36	0.36	1	0.28	0.055	0.35	0.35	0.75
Hongo [48]	78	0.26	0.21	0.28	0.35	1	0.21	0.04	0.26	0.26	0.79
Kobayashi [49]	6	0.22	− 0.22	0.16	0.26	− 0.42	0.34	− 0.34	0.18	0.37	− 0.46
Gough [50]	11	0.31	0.31	0.084	0.32	0.41	0.42	− 0.36	0.65	0.72	− 1.9
Timrot [51]	31	0.29	0.18	0.32	0.37	0.98	0.25	− 0.14	0.27	0.3	− 0.72
Carey^e^ [52]	43	0.93	0.8	1.4	1.6	6.8	0.73	0.58	1.3	1.4	6.9
Zozula^b,d^ [53]	79	0.77	− 0.27	0.95	0.98	2.6	1.5	− 1.2	1.5	1.9	− 4.7
Kestin [54]	33	0.11	0.095	0.097	0.13	0.33	0.1	0.025	0.14	0.14	0.39
Clarke [55]	13	0.46	0.46	0.21	0.51	0.94	0.27	− 0.15	0.43	0.44	− 1.2
Gracki [56]	46	0.63	− 0.63	0.37	0.73	− 1.8	0.64	− 0.63	0.52	0.81	− 2.3
Chierici [57]	12	0.63	− 0.63	0.44	0.76	− 1.4	0.58	− 0.58	0.39	0.68	− 1.6
Guevara [58]	22	0.48	− 0.17	0.51	0.53	− 0.96	0.33	0.14	0.38	0.4	0.78
Vermesse [59]	89	0.92	− 0.63	0.86	1.1	1.9	1.2	− 0.84	1.5	1.7	− 7.3
Kestin [60]	6	0.17	0.17	0.078	0.18	0.26	0.03	0.02	0.034	0.038	0.059
Kao [61]	35	0.63	− 0.52	0.58	0.77	− 2.3	0.75	− 0.62	0.74	0.96	− 2.8
Van Itterbeek [62]	38	2.2	0.17	2.5	2.5	− 4.3	2	− 0.68	2.4	2.5	− 5.3
Flynn [63]	34	0.3	− 0.21	0.33	0.39	− 0.89	0.29	− 0.18	0.29	0.33	− 0.85
Iwasaki [64]	32	0.13	0.11	0.13	0.17	0.38	0.12	− 0.0013	0.17	0.17	0.44
Van Itterbeek [65]	13	2.1	− 2.1	1.2	2.4	− 4.1	2.6	− 2.6	0.97	2.8	− 4.4
Kestin [66]	20	0.096	0.079	0.093	0.12	0.26	0.096	− 0.023	0.12	0.12	0.32
Kestin [67]	14	0.13	0.1	0.16	0.19	0.44	0.17	0.12	0.21	0.24	0.5
Kestin [68]	10	0.082	0.082	0.046	0.093	0.15	0.072	0.0043	0.093	0.088	0.17
Lazarre [69]	21	1.6	− 0.11	1.9	1.9	− 3.5	1.5	0.83	1.8	1.9	4.1
Rudenko [70]	8	1.2	− 0.32	1.5	1.4	− 2.5	1.2	− 0.72	1.3	1.5	− 2.4
Michels [71]	56	0.38	− 0.14	0.51	0.53	− 1.7	0.26	− 0.072	0.41	0.41	− 1.8
Overall	1719	0.62	− 0.082	1.2	1.2	− 9.8	1.1	− 0.3	3.2	3.2	33

^a^only points above 600 K included in primary data ^b^ used experimentally measured densities ^c^ point at 1597 K excluded ^d^ critical region data in Table 3 of Ref. [53] excluded Note that ref [33–35] do not appear in secondary as they have been replaced by ref. [32] ^e^ point at 624 K and 50.9 MPa excluded

#### Low-Pressure Gas Region (*P* < 1 MPa)

**Fig. 3:**
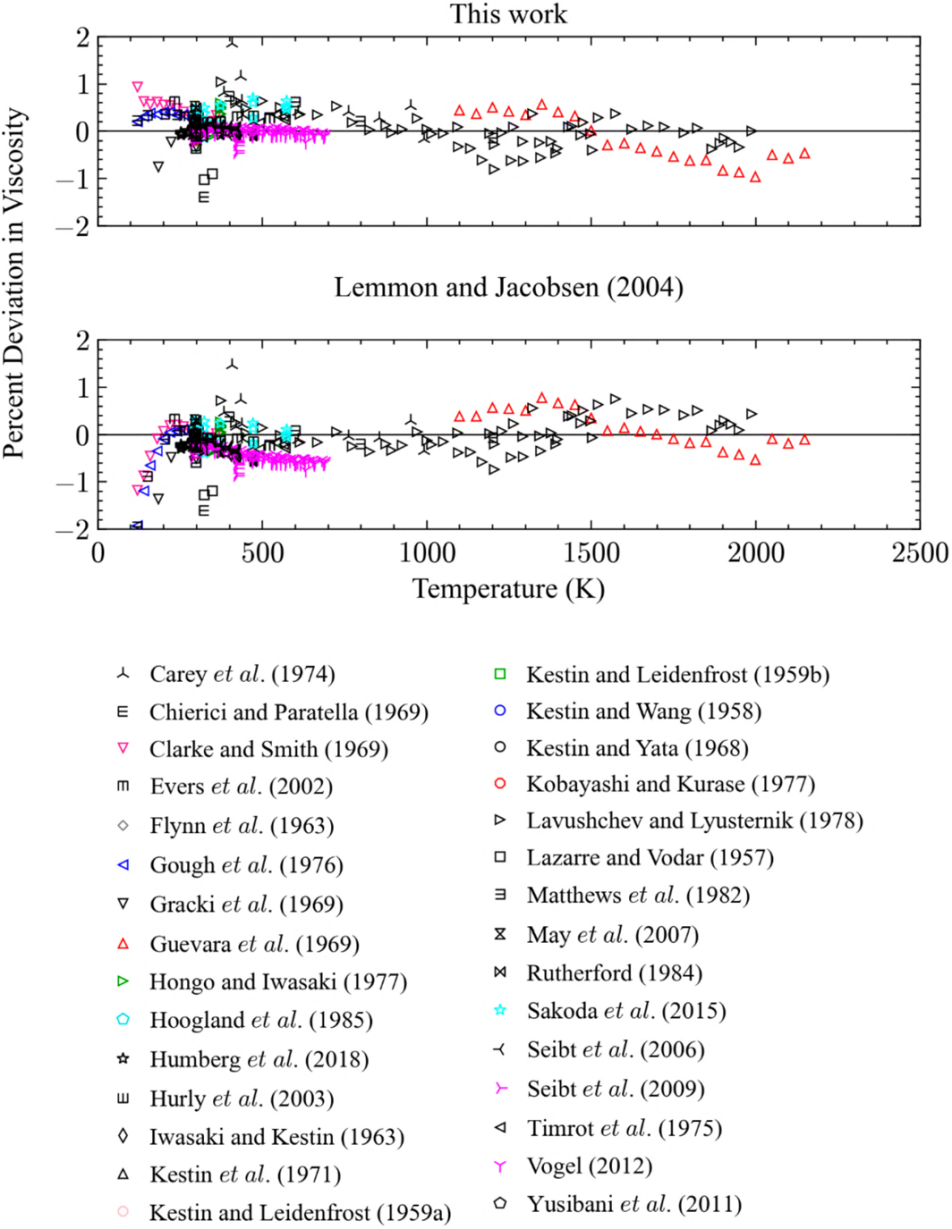
Percentage deviations of primary experimental data at low pressure calculated by the present model and the model of Lemmon and Jacobsen [7]

Comparisons with low-pressure gas, here defined as pressures less than 1 MPa, are shown in Fig. 3. The results between the present work and the correlation of Lemmon and Jacobsen [7] in this region are comparable, but a few notable exceptions are present. The first region is the region of low temperatures, below about 150 K. In this region, the Lemmon and Jacobsen model begins to show increasingly negative deviations from the data of Gough et al. [50], Clark and Smith [55], and Matthews et al. [46], due to the fact that they included the data of Lukin [84] and Johnston and McCloskey [132] in their fitting procedure. These two sets deviate from the theoretically based correlation of Xiao et al. [11–13] incorporated into our present model and distorted the Lemmon and Jacobsen [7] low-temperature results. Another significant difference concerns the representation of the data of Vogel [32] and Humberg et al. [29]. These two data sets have extremely low uncertainty, (0.15 % to 0.2 %) and (0.14 % to 0.19 %) respectively, with the higher uncertainty at the highest temperatures. These data cover the temperature range 253 K to 689 K, and the present correlation represents these data to within their uncertainties. The Lemmon and Jacobsen [7] model has an offset of about 0.3% to 0.5% with the correlation overpredicting the data. At the highest temperature of the Vogel [32] data, 589 K, the Lemmon and Jacobsen model is about 0.5 % too high. At temperatures above 689 K, the present model is based on the Xiao et al. [11–13] dilute-gas correlation and shows good agreement with the high-temperature experimental data of Lavuschchev and Lyusternik [47] and Guevara et al. [58].

##### The Second Viscosity Virial Coefficient

It is of interest to examine the behavior of the second viscosity virial coefficient. When the density is extremely low, the viscosity is a very weak function of density, and the expansion in Eq. 1 can be truncated to give,

11 $$\eta (\rho ,T) = \eta_{0} (T) + \eta_{1} (T)\rho ,$$

and the second viscosity virial coefficient *B*_*η*_ may be written as

12 $${\rm B}_{{\upeta }} ({\rm T}) = \frac{{\eta_{1} ({\rm T})}}{{\eta_{0} ({\rm T})}},$$

where *B*_*η*_ has units of L mol^−1^. If one has accurate low-density data along an isotherm, *B*_*η*_ can be calculated from the slope of the curve of viscosity vs. density using Eq. 12 and $\eta_{1} \, = \, \left( {\partial \eta /\partial \rho } \right)_{T}$ from Eq. 11.

**Fig. 4:**
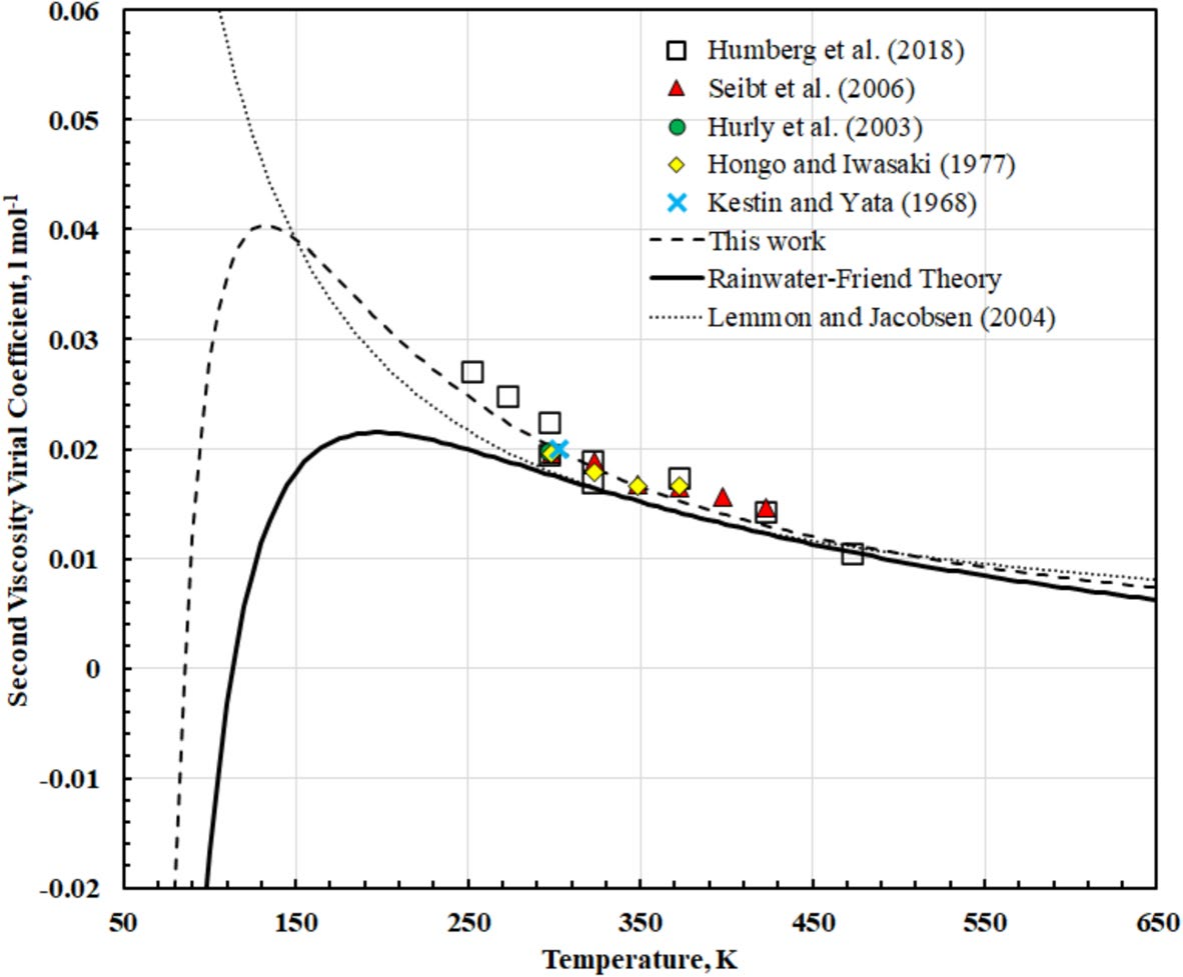
The second viscosity virial coefficient *B*_*η*_

Humberg et al. [29] derived values of *B*_*η*_ from their viscosity measurements along nine isotherms covering the temperature range 253.210 to 473.381 K. We assessed literature data and found several additional sources of high-quality viscosity data ([40, 41, 48, 60]) at very low density (less than 1 mol·L^−1^) along isotherms and used linear regression of the experimental data to obtain *B*_*η*_. These values are shown in Fig. 4, along with the present model, the Lemmon and Jacobsen [7] model, and Rainwater Friend theory using the correlation of Vogel et al. [147] with the parameters *σ* = 0.368 nm and *ε*/*k* = 90.9 K as recommended by Bich and Vogel [19]. The Lemmon and Jacobsen [7] correlation does not have the correct low-temperature behavior, as it continues to rise as the temperature is decreased. Our present model has the correct behavior at low temperatures and agrees with the literature data.

#### Liquid Region

**Fig. 5:**
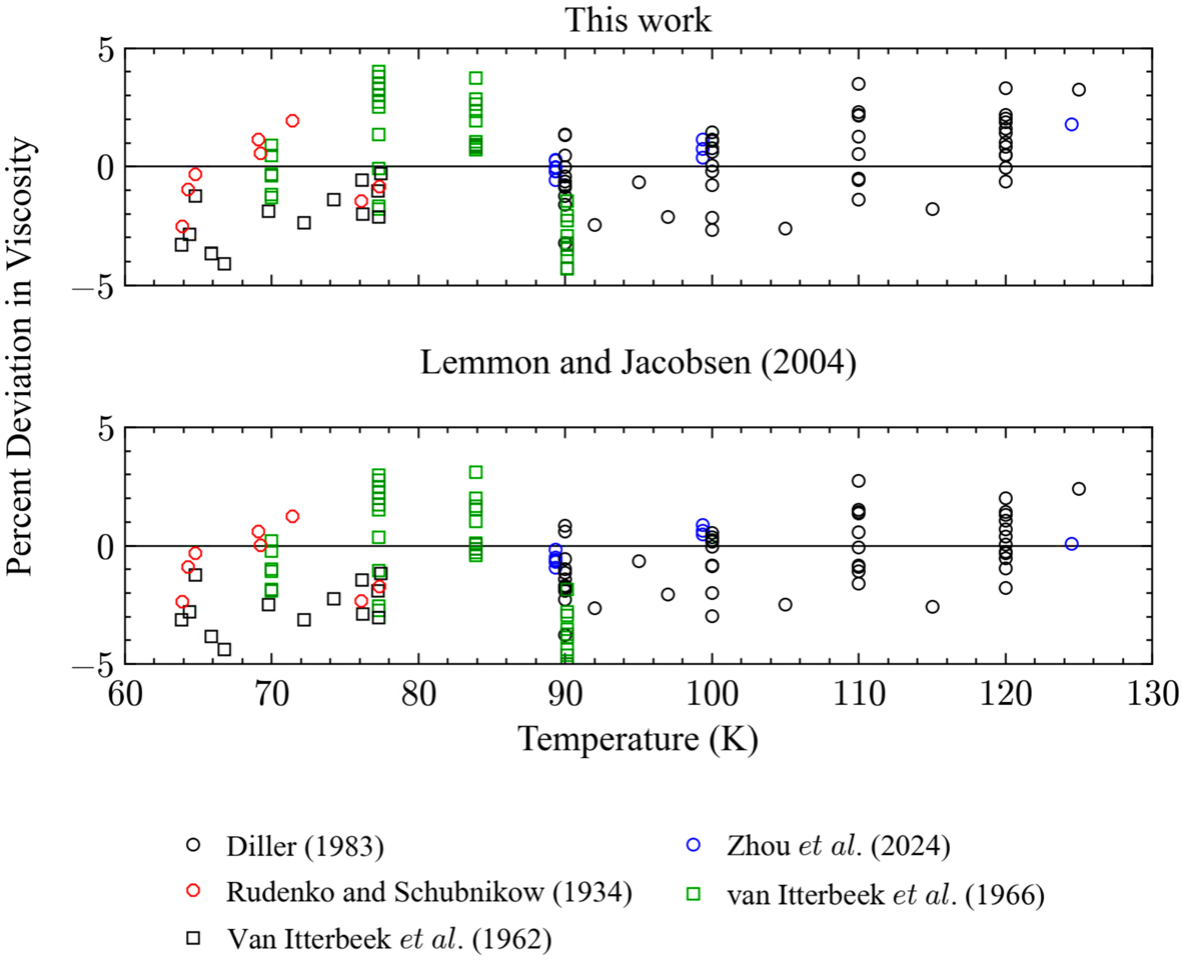
Percentage deviations of primary experimental data in the liquid phase as a function of temperature calculated by the present model and the model of Lemmon and Jacobsen [7]

**Fig. 6:**
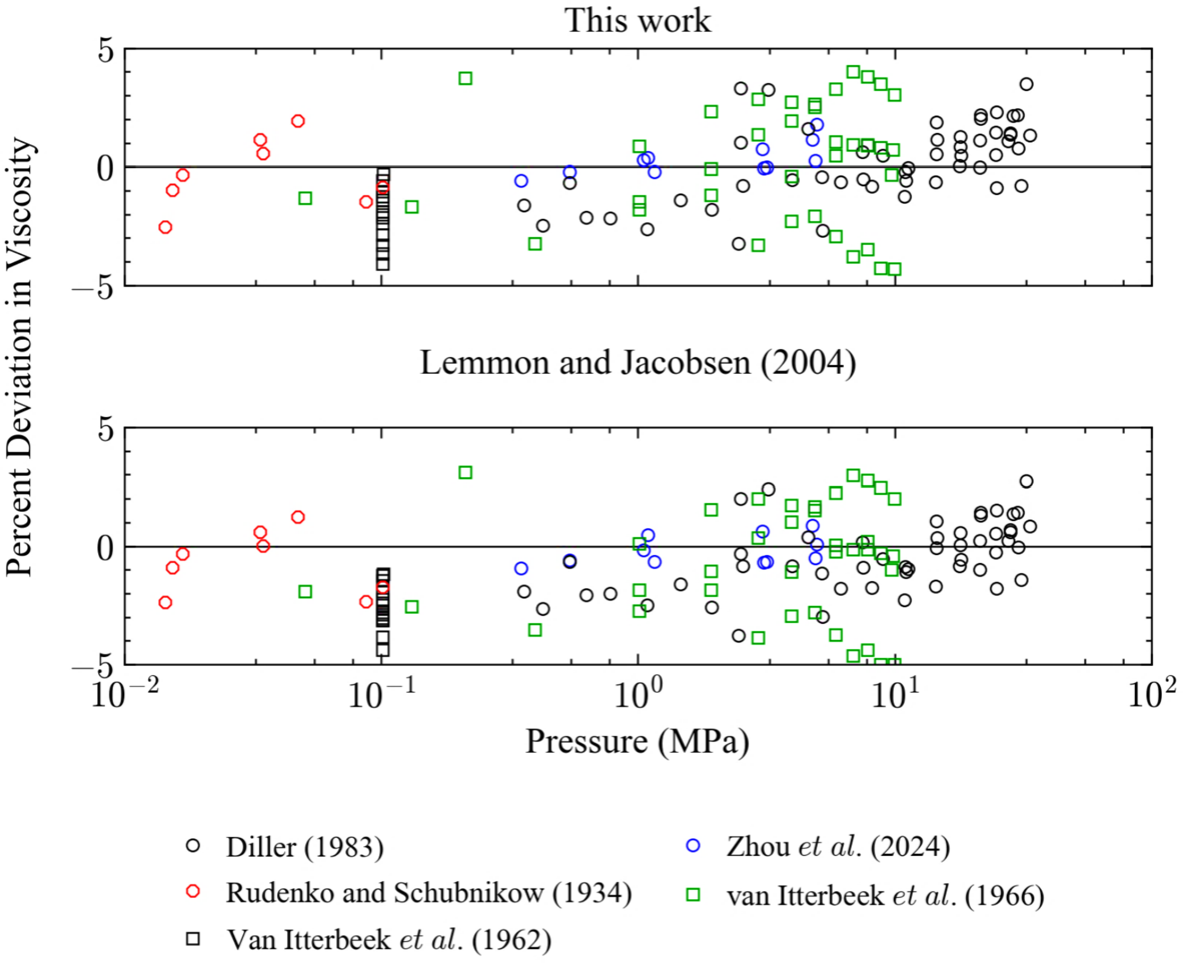
Percentage deviations of primary experimental data in the liquid phase as a function of pressure calculated by the present model and the model of Lemmon and Jacobsen [7]

Figures 5 and 6 show deviations for points in the liquid region as a function of *T* and *P*, respectively, where the liquid region is defined here as when the temperature is subcritical, and the density is greater than the critical density. The performance of the two models is very similar in this region and there are no significant differences; for the primary data in the liquid phase the AAD is 1.6 % for both models and they represent the data in the liquid region at pressures up to 34 MPa within 4 % (*k* = 2).

#### High-Pressure Region (*P* > 100 MPa)

**Fig. 7:**
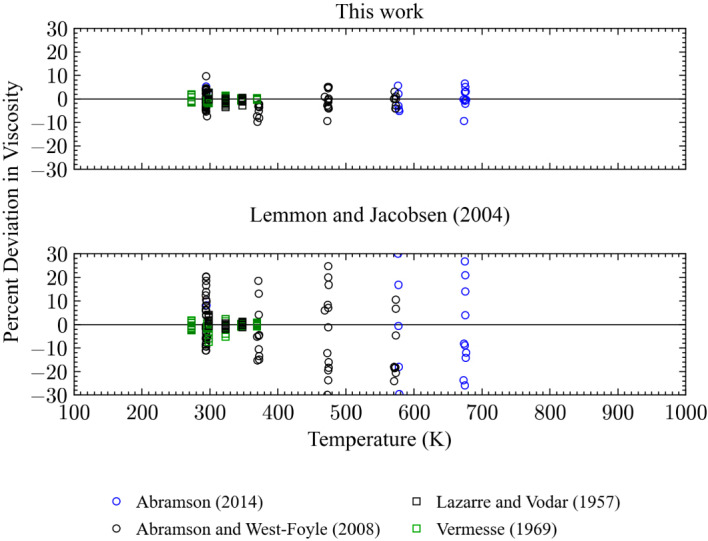
Percentage deviations of primary experimental data at high pressure as a function of temperature calculated by the present model and the model of Lemmon and Jacobsen [7]

**Fig. 8:**
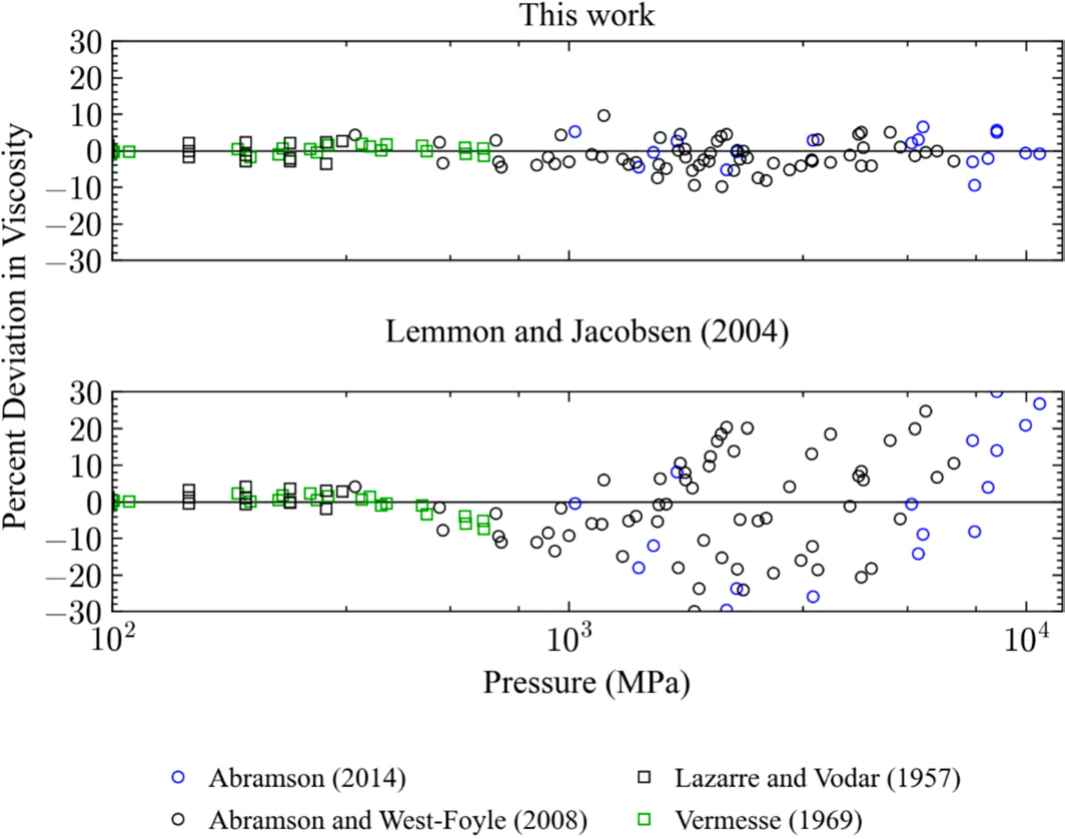
Percentage deviations of primary experimental data at high pressure as a function of pressure calculated by the present model and the model of Lemmon and Jacobsen [7]

Four sets of primary data that extend to pressures above 100 MPa; Abramson [31], Abramson and West-Foyle [38], Lazarre and Vodar [69], and Vermesse [59]. Comparisons with data above 100 MPa are shown in Figs. 7 and 8. It should be noted that the EOS of Span et al. [24] is validated only up to 2200 MPa and we are using the EOS in an extrapolation mode for pressures above 2200 MPa. The present model has deviations all below 10 %, while the Lemmon and Jacobsen model shows deviations above 10 %, reaching 30 % at pressures above about 700 MPa. However, the data sets of Abramson were not available at the time the Lemmon and Jacobsen correlation was made. The new data has permitted improvements in the representation of the viscosity at very high pressures.

#### Moderate-Pressure Region (1 MPa < *P* < 100 MPa)

**Fig. 9:**
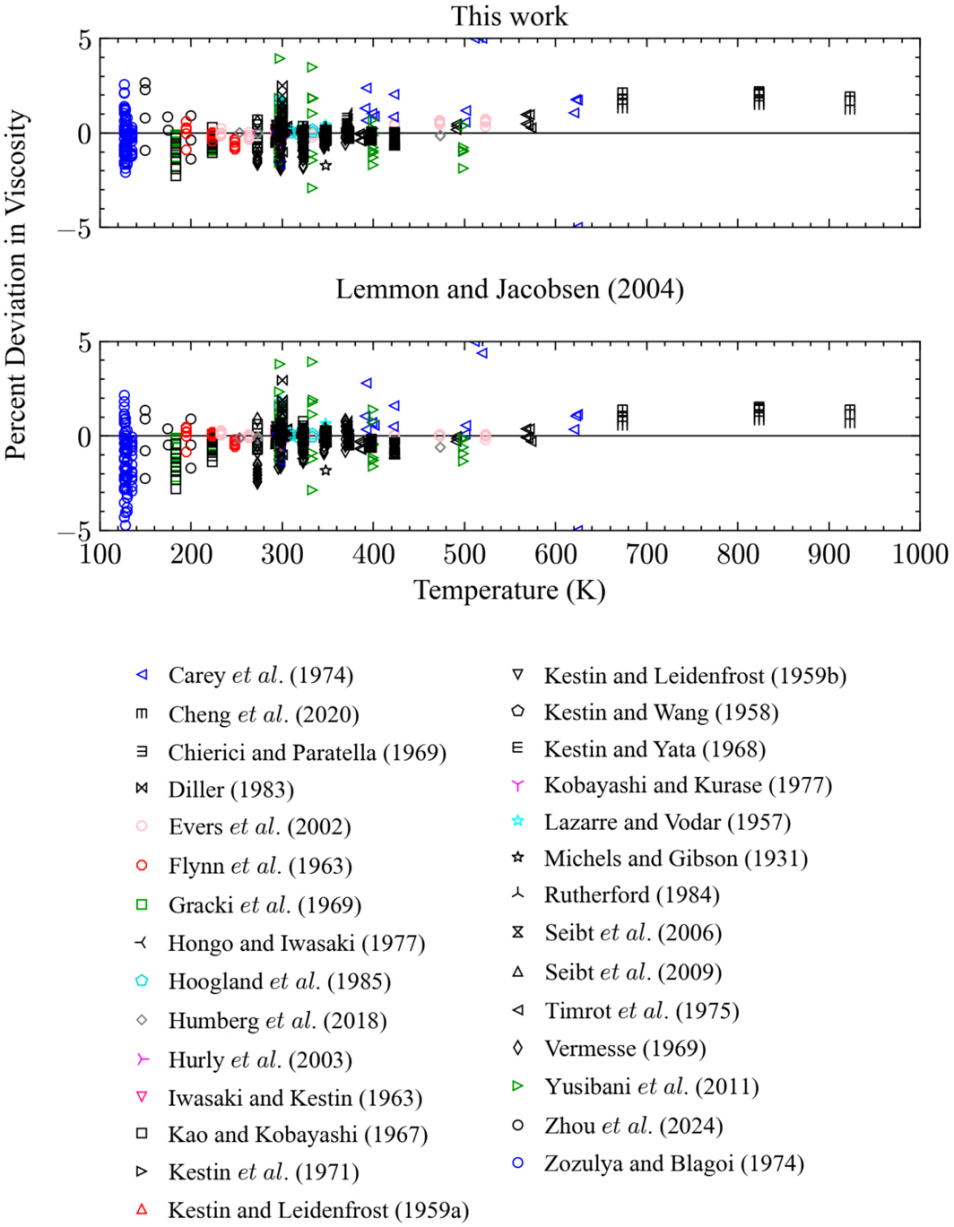
Percentage deviations of primary experimental data at moderate pressure in the supercritical region as a function of temperature calculated by the present model and the model of Lemmon and Jacobsen [7]

**Fig. 10:**
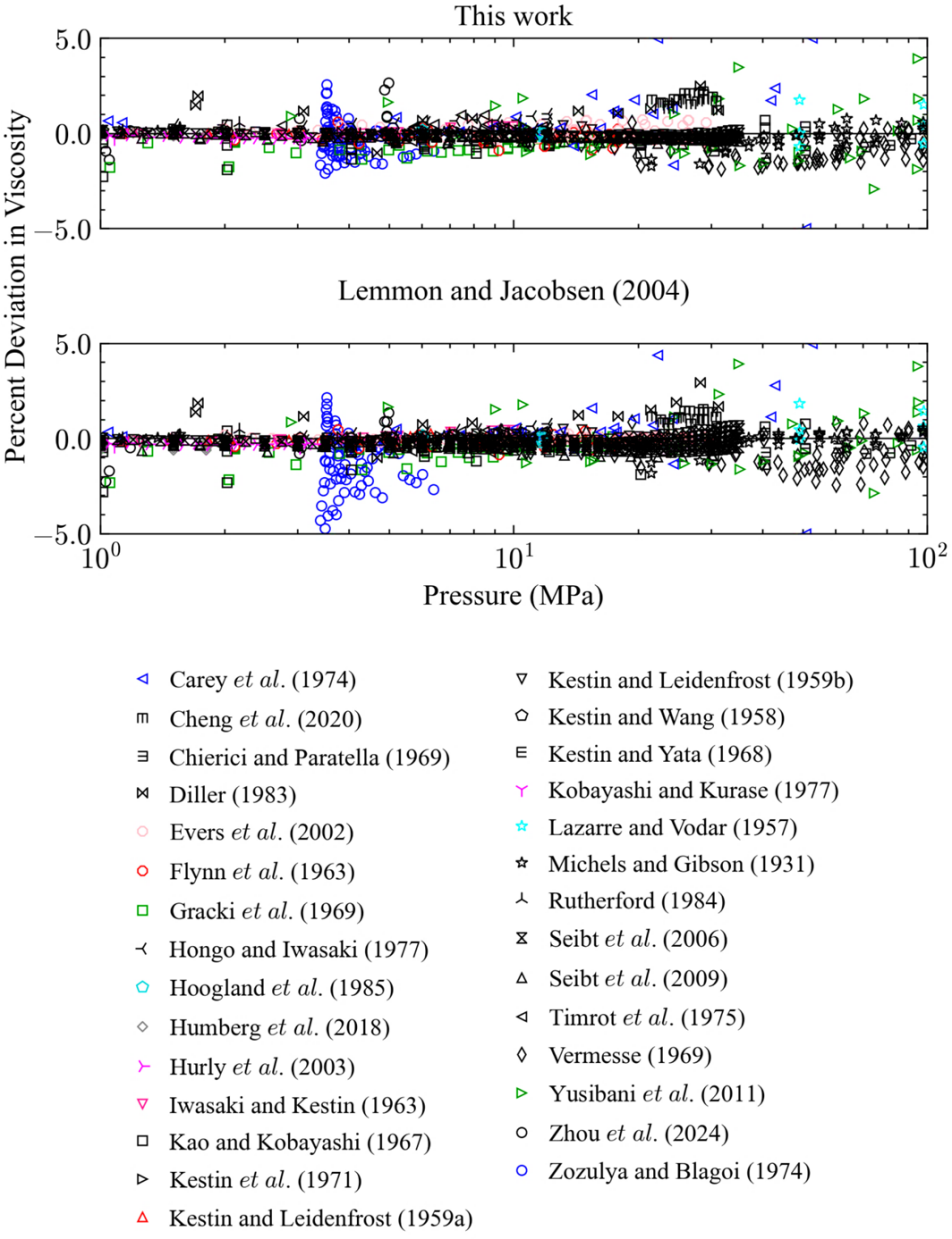
Percentage deviations of primary experimental data at moderate pressure in the supercritical region as a function of pressure calculated by the present model and the model of Lemmon and Jacobsen [7]

In the moderate pressure supercritical region (1 MPa < P < 100 MPa) there are data up to 923 K that are included in the primary data set and may be used for comparisons. In general, again the two models have similar performance, shown in Figs. 9 and 10. The standard deviation (*k* = 1) of all primary data in this range is 0.93 % for the Lemmon and Jacobsen model and 0.88 % for the present model. There are a few differences. The present model represents the data of Zozula et al. [53] better than the Lemmon and Jacobsen [7] model; the AAD and STDEV (*k* = 1) are 0.77 % and 0.98 % for the present model and 1.5 % and 1.9 % for the Lemmon and Jacobsen [7] model. These data are for four isotherms at 127 K, 128 K, 130 K, and 135 K in the region near the critical point and we gave them extra weight in order to represent them to within their experimental uncertainty (2 %) to provide a background viscosity that is consistent with additional data in the critical region by Zozula et al. [53] that is used in Sect. 2.4.5 to develop a model for the critical enhancement. As shown in Fig. 10, the data of Vermesse [59] also are represented slightly better in the present model, with an AAD and STDEV of 0.92 % and 0.86 % for the present model and 1.2 % and 1.5 % for the Lemmon and Jacobsen [7] model. The Lemmon and Jacobsen model does a better job at representing the data of Evers et al. [42], probably because the present correlation includes the data of Seibt et al. [40] in this region. The Lemmon and Jacobsen model also has a better representation of the recent data of Zhou et al. [27] although the present model represents the data to within its uncertainty, which is fairly large, 4.2 %. Additional comparison plots for the moderate pressure region are given in the Supplemental Information.

#### Critical Region

The critical enhancement contribution for the viscosity of nitrogen follows the successful application of the crossover theory of Bhattacharjee and coworkers [150, 151] to carbon dioxide [21], ethane [22], water [20, 152], heavy water [153], xenon [154, 155], and ethylene [16]. The analysis of the critical enhancement contribution is based on the 1974 data set of Zozulya and Blagoi [53] in the critical region. The critical enhancement from this data set was also considered in earlier analysis in 1979 by Basu and Sengers [148, 149] based on the theory of viscosity enhancement in the asymptotic scaling limit. This asymptotic scaling approach is only valid very close to the critical point and in 1981 Bhattacharjee et al. [151] demonstrated the validity of the same crossover function for the critical enhancement of viscosity that we apply here, for both nitrogen and water. The data and previous analysis were based on the IPTS-68 temperature scale and the correlation length did not account for the background compressibility. In the present work, we calculate the correlation length from the equation of state [24] as described below and the viscosity data have been corrected to the current values for the critical temperature of nitrogen [24] on ITS-90.

The critical enhancement is a function of the correlation length, *ξ*, which can be calculated from the equation of state as described below. The critical enhancement term is given by:

13 $$\, \Delta \eta_{{\text{c}}} { = }\;{\text{exp}}\left( {x_{\mu } Y} \right),$$

where *x*_*μ*_ is a universal dynamic critical exponent, for which we have adopted the most recent theoretical value of 0.068 [156]. The crossover function *Y* is defined by [151]

14 $$\begin{gathered} Y = \frac{1}{12}\sin \left( {3\psi_{{\text{D}}} } \right) - \frac{1}{{4q_{{\text{C}}} \xi }}\sin \left( {2\psi_{{\text{D}}} } \right) + \frac{1}{{\left( {q_{{\text{C}}} \xi } \right)^{2} }}\left[ {1 - \frac{5}{4}\left( {q_{{\text{C}}} \xi } \right)^{2} } \right]\sin \left( {\psi_{{\text{D}}} } \right) \hfill \\ \quad \quad - \frac{1}{{\left( {q_{{\text{C}}} \xi } \right)^{3} }}\left\{ {\left[ {1 - \frac{3}{2}\left( {q_{{\text{C}}} \xi } \right)^{2} } \right]\psi_{{\text{D}}} - \left| {\left( {q_{{\text{C}}} \xi } \right)^{2} - 1} \right|^{3/2} L(w)} \right\}, \hfill \\ \end{gathered}$$

with

15 $$\psi_{{\text{D}}} = \arccos \;\left[ {\left( {1 + q_{{\text{D}}}^{2} \xi^{2} } \right)^{{{\raise0.7ex\hbox{${ - 1}$} \!\mathord{\left/ {\vphantom {{ - 1} 2}}\right.\kern-0pt} \!\lower0.7ex\hbox{$2$}}}} } \right]\,,$$

and with the function *L*(*w*) given by.

16 $$L(w) = \left\{ {\begin{array}{cc} {\ln \frac{1 + w}{{1 - w}}\,,\quad \quad {\text{for}}\;q_{{\text{C}}} \xi > 1} \\ {{2}\;{\text{arctan}}\,\left| w \right|\,,\quad {\text{for}}\;q_{{\text{C}}} \xi \le 1} \\ \end{array} } \right\}.$$

The variable *w* is defined as

17 $$w = \left| {\frac{{q_{{\text{C}}} \xi - 1}}{{q_{{\text{C}}} \xi + 1}}} \right|^{{{\raise0.7ex\hbox{$1$} \!\mathord{\left/ {\vphantom {1 2}}\right.\kern-0pt} \!\lower0.7ex\hbox{$2$}}}} \tan \left( {\frac{{\psi_{{\text{D}}} }}{2}} \right).$$

The function *Y* contains two system-dependent constants, namely, the wave numbers *q*_C_ and *q*_D_.

For small *ξ*, the function *Y* approaches zero, so that *η* approaches the background viscosity *η*_b_ (where there is no enhancement) in this limit. Around *ξ* = 0, the function for *Y* has a Taylor expansion of the form,

18 $$Y = \frac{1}{5}q_{{\text{C}}} \xi \;\left( {q_{{\text{D}}} \xi } \right)^{5} \left( {1 - q_{{\text{C}}} \xi + \left( {q_{{\text{C}}} \xi } \right)^{2} - \frac{765}{{504}}\left( {q_{{\text{D}}} \xi } \right)^{2} } \right).$$

This expansion expression should be used for 0 ≤ *ξ* ≤ 0.06 nm.

The wave number *q*_C_ is related to a background contribution to the decay rate of the critical fluctuations and is given by

19 $$q_{{\text{C}}} = \frac{{k_{{\text{B}}} T_{{\text{c}}}^{2} }}{{16\eta_{{\text{b}}}^{{\text{c}}} \lambda_{{\text{b}}}^{{\text{c}}} P_{{\text{c}}} }}\frac{{\Gamma_{0} }}{{\xi_{0}^{2} }}\left( {\frac{\partial P}{{\partial T}}} \right)_{{\rho = \rho_{{\text{c}}} }}^{2} ,$$

where *k*_B_ is Boltzmann’s constant and where $\eta_{{\text{b}}}^{{\text{c}}}$ and $\lambda_{{\text{b}}}^{{\text{c}}}$ are the values of the background viscosity and background thermal conductivity, respectively, at the critical point, while $(\partial P/\partial T)_{{\rho = \rho_{{\text{c}}} }}$ is the slope of the critical isochore at the critical temperature, and *ξ*_0_ and Γ_0_ are the amplitudes of the asymptotic power laws. We use the term background to encompass the region where the critical enhancement is negligible. The wave number *q*_D_ represents a Debye cutoff of the mode-coupling integrals for critical dynamics and is the only adjustable parameter in the theory.

The correlation length is given by

20 $$\xi = \xi_{0} \left( {\frac{{\Delta \overline{\chi } }}{{\Gamma_{0}^{{}} }}} \right)^{{{\raise0.7ex\hbox{$\nu $} \!\mathord{\left/ {\vphantom {\nu \gamma }}\right.\kern-0pt} \!\lower0.7ex\hbox{$\gamma $}}}} ,$$

in terms of $\Delta \overline{\chi }$ (≥ 0), which is defined by.

21 $$\Delta \overline{\chi } = \left[ {\overline{\chi } \left( {\overline{T} ,\overline{\rho } } \right) - \overline{\chi } \left( {\overline{T}_{{\text{R}}} ,\overline{\rho } } \right)\frac{{\overline{T}_{{\text{R}}} }}{{\overline{T} }}} \right].$$

In Eq. 20*γ* and *ν* are universal critical exponents for which *ν* = 0.630 and *γ* = 1.239 [157], and the amplitudes for the correlation length *ξ* of nitrogen are

22 $$\xi_{0} = 0.16\;{\text{nm,}}\;\Gamma_{{0}} = 0.075\;,$$

as given in Bhattacharjee et al. [151]. In Eq. 21, $\overline{T}_{{\text{R}}}$ = 1.5, is a reference temperature sufficiently high above the critical temperature where the critical fluctuations can be assumed to be small. The susceptibility *χ* is related to the isothermal compressibility, $\chi = \rho (\partial \rho /\partial P)_{T}$ [158] with its dimensionless form expressed as $\overline{\chi }$ = *P*_c_*χ/ρ*_c_^2^. Furthermore, $\Delta \overline{\chi }$ is to be taken to be zero when the right-hand side of Eq. 21 becomes negative.

The critical parameters of the reference equation of state for nitrogen are [24]

$$T_{{\text{c}}} \, = \,126.192 {\text{K}},\rho_{{\text{c}}} \, = \,11.1839 {\text{mol}} \cdot {\text{L}}^{{ - 1}} ,P_{{\text{c}}} \, = \,3.3958 {\text{MPa}}.$$

The wavenumber *q*_C_ is given by Eq. 19 with the relevant properties for nitrogen. From the previous correlation for the thermal conductivity of nitrogen [7], it is found that $\lambda_{b}^{c} = 31.8\;{\text{mW}} \cdot {\text{m}}^{{ - 1}} \cdot {\text{K}}^{{ - 1}}$, while the present correlation for the background viscosity of nitrogen gives $\eta_{{\text{b}}}^{{\text{c}}}$ = 18.25 Paμ·s. From the equation of state [24], it is found that$\left( {\partial P/\partial T} \right)_{{\rho = \rho_{{\text{c}}} }} = 0.1644\; {\text{MPa}}\cdot{\text{K}}^{{ - {1}}}$. The properties for nitrogen are substituted into Eq. 19 to calculate $q_{{\text{C}}}^{ - 1} = 1.81\;{\text{nm}}\,.$ All information required for calculation of the critical viscosity enhancement is now available, except for the system-dependent wave number *q*_D_ which is optimized to best fit the viscosity data in the critical region. The earlier analysis of Bhattacharjee et al. [151] found that *q*_D_^−1^ = 1.2 nm was optimum for their analysis. We find better representation with *q*_D_^−1^ = 0.8 nm for the present background viscosity and equation of state [24].

Zozulya and Blagoi [53] report viscosity data in Tables I and II for 5 isotherms as a function of temperature and density, and in Table III report additional data for an additive critical enhancement *η*_c_ as a function of temperature difference from the critical temperature and density for 6 additional isotherms where they observed critical enhancement. Thus, their additive viscosity critical enhancement *η*_c_ is summed with the value calculated with the present correlation for background viscosity according to [53]

24 $$\eta \,\left( {\rho ,{\rm T}} \right)\, = \,\eta_{0} {\kern 1pt} \left( {\rm T} \right)\, + \,\Delta \eta_{{{\text{res}}}} \left( {\rho ,{\rm T}} \right)\, + \eta_{{\text{c}}} {\kern 1pt} \left( {\rho {,}{\rm T}} \right).$$

They reported that their best estimate for the critical temperature based on their measurements was 126.21 K (IPTS-68) [53]. The current critical temperature for nitrogen based on the equation of state is 126.192 K (ITS-90) [24]. Zozulya and Blagoi [53] reported data along the critical isotherm, which would now be 126.192 K. Looking at the data along the critical isotherm we believe that the isotherm temperature needs to be adjusted to 126.194 K. This becomes the reference temperature for all the data of Zozulya and Blagoi [53]. For our analysis of the critical region, the viscosity isotherms of Zozulya and Blagoi are at 126.194 K, 126.984 K, 127.984 K, 129.984 K, and 134.984 K. The additional critical enhancement isotherms are at 126.224 K, 126.244 K, 126.284, 126.384 K, 126.584 K, and 126.984 K. At 126.984 K, there are both viscosity data and critical enhancement data reported. The reported viscosity data are consistent with the viscosity data based on Eq. 24 and the present background viscosity correlation at this temperature.

**Fig. 11:**
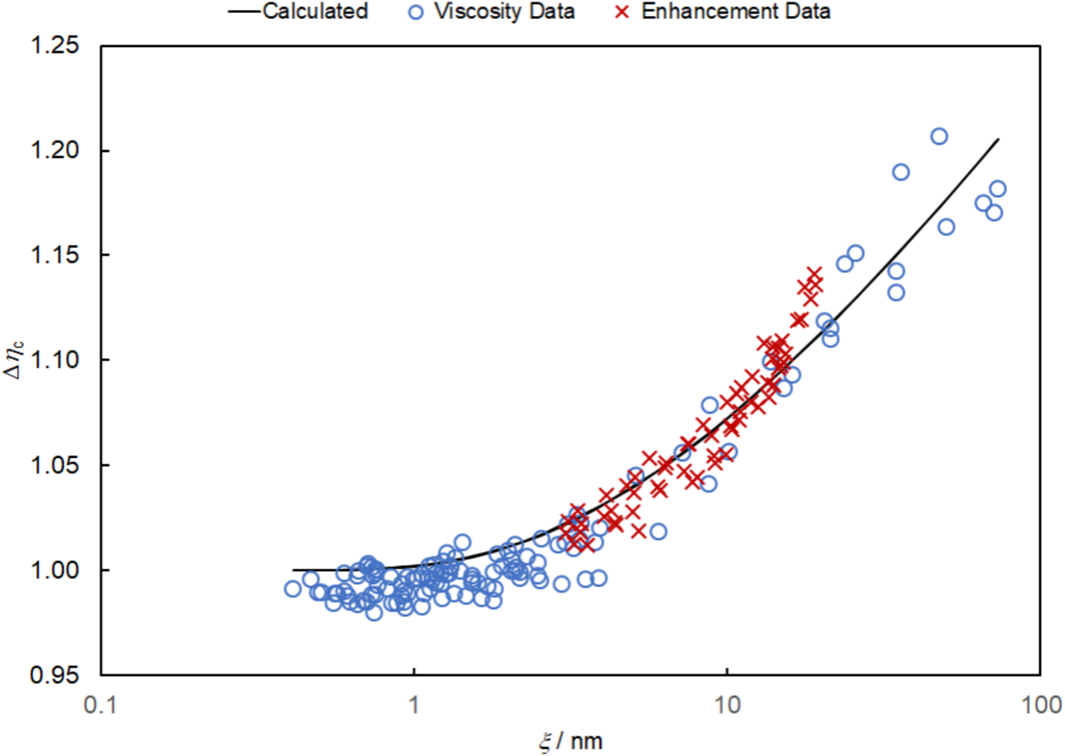
The critical enhancement calculated with Eqs. 13–21 is shown with the experimental results from viscosity isotherms (+) and from critical enhancement isotherms ( ×) of Zozulya and Blagoi [53]. The critical enhancement is a function of the correlation length calculated from the equation of state

Figure 11 targetshows the critical enhancement calculated with Eqs. 13–21 and the critical enhancement data of Zozulya and Blagoi [53] as a function of the calculated correlation length.

Good agreement is found between the calculations for the critical enhancement and the data of Zozulya and Blagoi [53] in the critical region. The only adjustable parameter in the critical enhancement calculation is *q*_D_^−1^, and its optimum value is found to 0.8 nm.

**Fig. 12:**
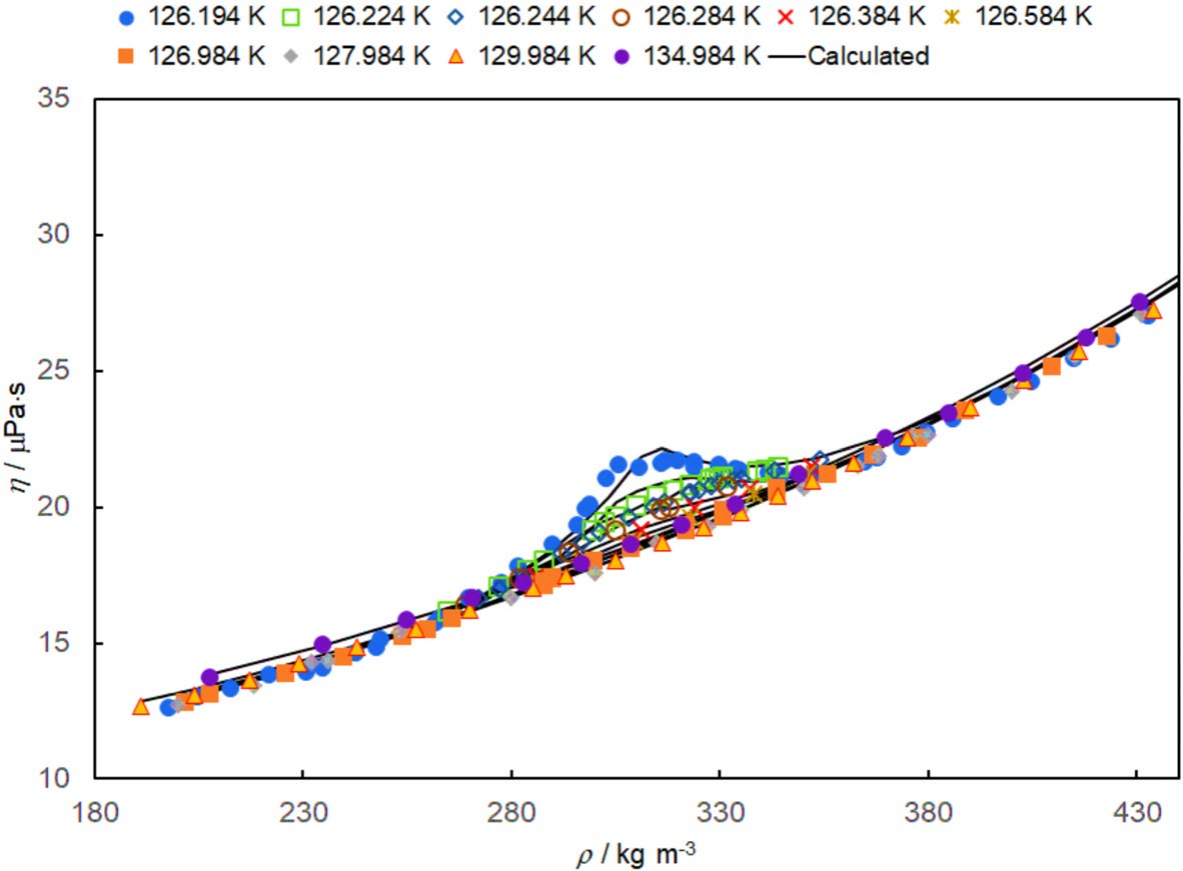
The viscosity isotherms of Zozulya and Blagoi [53] are shown as a function of density. The filled symbols designate the reported viscosity data, while the open symbols designate viscosity derived from the reported additive critical enhancement. Solid lines represent the calculated viscosity from the present correlation for each of these isotherms

The reported viscosity in the critical region and the full correlation including the critical enhancement are shown in Fig. 12. The full correlation provides a good representation of the data of Zozulya and Blagoi [53] in the critical region.

**Fig. 13:**
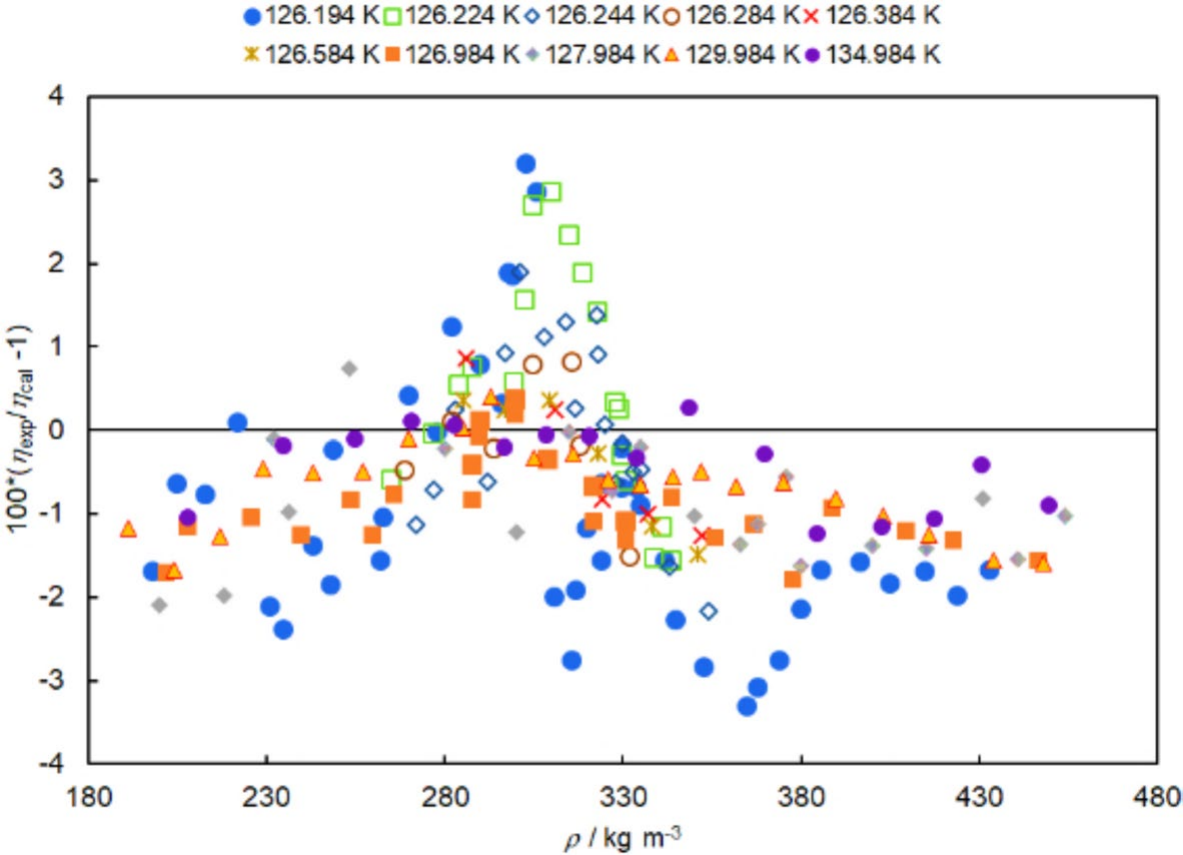
Deviations between the viscosity isotherms of Zozulya and Blagoi [53] and the correlation reported here are shown as a function of density. The filled symbols designate the reported viscosity data, while the open symbols designate viscosity derived from the reported additive critical enhancement

The relative deviations between the data and the viscosity values calculated with the full correlation developed here are shown in Fig. 13. Deviations between the experimental values and the correlation are generally found to be within ± 3 %. This should be considered relative to the relative magnitude of the critical enhancement of viscosity found in these data in the critical region, which is a maximum of 20% (∆*η*_c_ of 1.20). Since the critical enhancement of viscosity is significant only very close to the critical point, it becomes very sensitive to uncertainty in both temperature and density. Thus, the agreement between the data of Zozulya and Blagoi [53] and the crossover critical enhancement theory of Bhattacharjee et al. [151] is considered very good based on our analysis. This conclusion agrees with the previous work of Bhattacharjee et al. [151].

#### Secondary Data

Table 6 presents comparisons of the primary data with the present correlation, (Eqs. 1,3,5) and with the correlation of Lemmon and Jacobsen [7]. The critical enhancement term Δ*η*_c_ is set to one for these comparisons.

**Table 6:** Evaluation of the nitrogen viscosity correlation Eqs. 1,3,5 for the secondary data

		Present model					Lemmon and Jacobsen (2004) model [7]				
First author	NPTS	AAD	BIAS	STDEV	RMS	MAX	AAD	BIAS	STDEV	RMS	MAX
Pinho [72]	1	14	14	–	14	14	14	14	–	14	14
Lv [73]	24	0.23	0.16	0.21	0.26	0.51	0.19	0.065	0.24	0.24	− 0.49
Wang [74]	24	0.29	− 0.037	0.36	0.36	-0.7	0.3	-0.13	0.35	0.37	-0.8
El Hawary [75]	116	0.24	0.16	0.25	0.29	0.77	0.11	− 0.023	0.14	0.14	0.45
Tomida [76]	30	0.66	0.66	0.091	0.67	0.88	0.37	0.37	0.11	0.38	0.65
Sih [77]	20	2.1	− 0.22	2.6	2.6	− 4.7	2.2	− 0.28	2.7	2.6	− 4.8
Audonnet [78]	8	4.2	− 4.1	4.1	5.6	− 11	4.2	− 4.1	4.1	5.6	− 11
Assael [79]	7	0.53	0.5	0.44	0.64	1.1	0.37	0.16	0.47	0.47	0.9
Docter [80]	31	0.41	0.41	0.2	0.45	0.96	0.38	0.19	0.42	0.46	1.2
Dunlop [81]	1	0.2	0.2	–	0.2	0.2	0.027	− 0.027	–	0.027	− 0.027
Hansen [82]	6	1.1	− 0.0004	1.4	1.3	1.9	1.1	− 0.34	1.3	1.3	− 2.2
Strehlow [83]	47	0.19	0.19	0.19	0.27	1.4	0.29	− 0.23	0.26	0.34	1.2
Lukin [84]	23	0.95	0.89	1.1	1.4	3.7	0.21	− 0.21	0.096	0.23	− 0.43
Kestin [85]	5	0.37	0.37	0.13	0.39	0.55	0.05	0.032	0.068	0.068	0.14
Abe [86]	5	0.59	0.59	0.3	0.65	0.97	0.29	0.26	0.31	0.38	0.7
Kestin [87]	9	0.61	0.61	0.3	0.67	1	0.3	0.22	0.34	0.38	0.7
Kestin [88]	9	0.42	0.42	0.19	0.46	0.66	0.2	0.16	0.26	0.29	0.65
Matthews [89]	15	0.81	− 0.14	1	0.97	− 1.9	0.68	− 0.51	0.7	0.85	− 1.5
Schlumpf [90]	11	0.9	− 0.35	1.1	1.1	− 1.8	0.52	0.46	0.55	0.7	1.4
Golubev [91]	70	2.5	− 2.5	1.9	3.1	− 7.9	1.9	− 1.6	2.2	2.7	− 8.7
Maitland [92]	24	0.91	− 0.55	0.96	1.1	− 1.8	0.84	− 0.68	0.73	0.99	− 1.8
Borisov [93]	1	0.29	0.29	–	0.29	0.29	0.057	0.057	–	0.057	0.057
Hellemans [94]	6	0.59	0.59	0.29	0.64	1	0.21	0.17	0.27	0.3	0.54
Kestin [95]	8	0.43	0.43	0.28	0.5	0.85	0.17	0.055	0.22	0.21	0.4
Kestin [96]	6	0.44	0.44	0.21	0.48	0.7	0.14	0.098	0.14	0.16	0.26
Golubev [97]	4	1.1	− 0.97	0.9	1.2	− 1.8	1.4	− 1.4	1	1.6	− 2.3
Dawe [98]	15	0.74	− 0.45	0.76	0.86	− 1.5	0.67	− 0.59	0.51	0.77	− 1.1
Grevendonk [99]	134	3.4	2.5	3.8	4.5	13	3.2	1.7	3.9	4.2	13
Hellemans [100]	44	8.4	− 8.1	6.6	10	− 26	9.1	− 8.9	7.1	11	− 27
Hellemans [101]	18	31	− 31	24	39	− 76	32	− 32	24	40	− 78
Munczak [102]	3	1.8	1.8	2.5	2.7	4.6	1.5	1.5	2.5	2.5	4.4
Timrot [103]	8	0.73	0.73	0.38	0.81	1.3	0.34	0.31	0.34	0.44	0.82
Clarke [104]	12	0.39	0.28	0.32	0.42	0.61	0.59	− 0.41	0.99	1	− 3
DiPippo [105]	41	0.53	0.53	0.28	0.6	1	0.22	0.18	0.19	0.26	0.49
Shepeleva [106]	64	4.5	2.4	8.1	8.4	36	4.5	1.7	8.3	8.4	35
Boon [107]	4	9.7	− 9.7	0.32	9.7	− 10	10	− 10	0.28	10	− 11
DiPippo [108]	5	0.13	0.13	0.03	0.13	0.17	0.032	− 0.016	0.04	0.04	− 0.061
Gururaja [109]	2	0.18	0.18	0	0.18	0.18	0.048	− 0.048	0	0.048	− 0.048
DiPippo [110]	24	0.55	0.49	0.37	0.61	0.98	0.26	0.15	0.29	0.32	− 0.61
Reynes [111]	30	2.1	− 2.1	1	2.3	− 5.4	2	− 2	0.96	2.2	− 5.2
Rigby [112]	15	1.6	− 1.5	1	1.8	− 3.5	2	− 2	1	2.2	− 3.7
Van Itterbeek [62]	33	1.8	1.6	1.5	2.2	5.6	1.3	0.92	1.5	1.7	5.2
Forster [113]	10	11	11	5.9	12	20	11	11	5.9	12	19
Goldman [114]	16	1.1	0.76	1.5	1.6	4.5	1.1	0.85	1.5	1.7	4.7
Kestin [115]	37	1.2	1.2	0.71	1.4	2.7	0.81	0.76	0.63	0.98	2.1
Makavetskas [116]	62	3.5	3.3	3.2	4.5	12	3.3	3	3.4	4.5	12
Vermesse [117]	24	1.8	0.47	2	2	3.6	2.1	0.88	2.4	2.5	5
Filippova [118]	27	8	− 7.3	6.6	9.8	− 20	8.7	− 8.2	7	11	− 21
Baron [119]	40	1.2	0.65	1.3	1.5	3.1	1.4	0.72	1.5	1.7	3.8
Ellis [120]	7	5.4	− 5.4	0.86	5.4	− 6.3	5.4	− 5.4	0.75	5.4	− 6.1
Glaser [121]	124	4.7	− 1.5	5.7	5.8	13	4.6	− 1.7	5.5	5.7	13
Makita [122]	54	2.5	− 2.2	3.6	4.2	− 11	2.4	− 2.1	3.6	4.2	− 11
Ross [123]	41	2.3	− 1.9	2.9	3.4	− 9.3	2.5	− 2.1	3.3	3.9	− 11
Kiyama [124]	24	0.58	− 0.24	0.69	0.72	− 1.6	0.43	− 0.1	0.56	0.55	− 1.3
Iwasaki [125]	25	0.45	− 0.25	0.52	0.56	− 1.5	0.56	− 0.44	0.6	0.74	− 1.9
Golubev [126]	27	1.6	− 0.18	2	2	− 4.5	1.1	− 1	1.5	1.8	− 4.6
Bonilla [127]	25	4.7	− 4.6	4.1	6.1	− 14	4.6	− 4.6	3.8	5.9	− 14
Buddenberg [128]	6	0.13	− 0.11	0.14	0.17	− 0.36	0.34	− 0.34	0.14	0.36	− 0.59
Schmid [129]	11	1.9	− 1	2.2	2.4	− 4.5	1.9	− 1.3	2.1	2.3	− 4.4
Wobser [130]	5	0.3	− 0.3	0.13	0.32	− 0.47	0.58	− 0.58	0.17	0.59	− 0.8
Gerf [131]	7	2.9	− 2.8	2.8	3.8	− 7.1	3.5	− 3.5	2.6	4.3	− 7.3
Johnston [132]	16	1	1	0.93	1.4	3.5	0.2	0.15	0.19	0.24	0.46
Rudenko [133]	7	16	16	13	20	29	17	16	13	20	29
Herning [134]	1	0.44	0.44	–	0.44	0.44	0.21	0.21	–	0.21	0.21
Trautz [135]	9	1.1	− 1.1	0.52	1.2	− 1.9	1.5	− 1.5	0.63	1.7	− 2.4
Boyd [136]	68	5.7	5.7	3.8	6.8	18	5.7	5.7	3.8	6.8	18
Trautz [137]	4	1.1	− 1.1	0.51	1.2	− 1.6	1.5	− 1.5	0.63	1.6	− 2.2
Trautz [138]	12	2.8	− 2.8	1.4	3.1	− 5.3	3.2	− 3.2	1.3	3.4	− 5.4
Smith [139]	2	0.55	0.55	0.21	0.57	0.7	0.28	0.28	0.14	0.3	0.38
Yen [140]	1	0.13	− 0.13	–	0.13	− 0.13	0.36	− 0.36	–	0.36	− 0.36
Vogel [141]	2	1.4	1.4	0.34	1.4	1.6	1.2	− 0.33	1.8	1.3	− 1.6
Schmitt [142]	6	0.61	0.61	0.23	0.65	0.88	0.28	0.28	0.17	0.32	0.55
Markowski [143]	30	0.69	0.69	0.9	1.1	5.1	0.43	0.36	0.89	0.95	4.8

### Range and Uncertainty Estimates for the Correlation

**Fig. 14:**
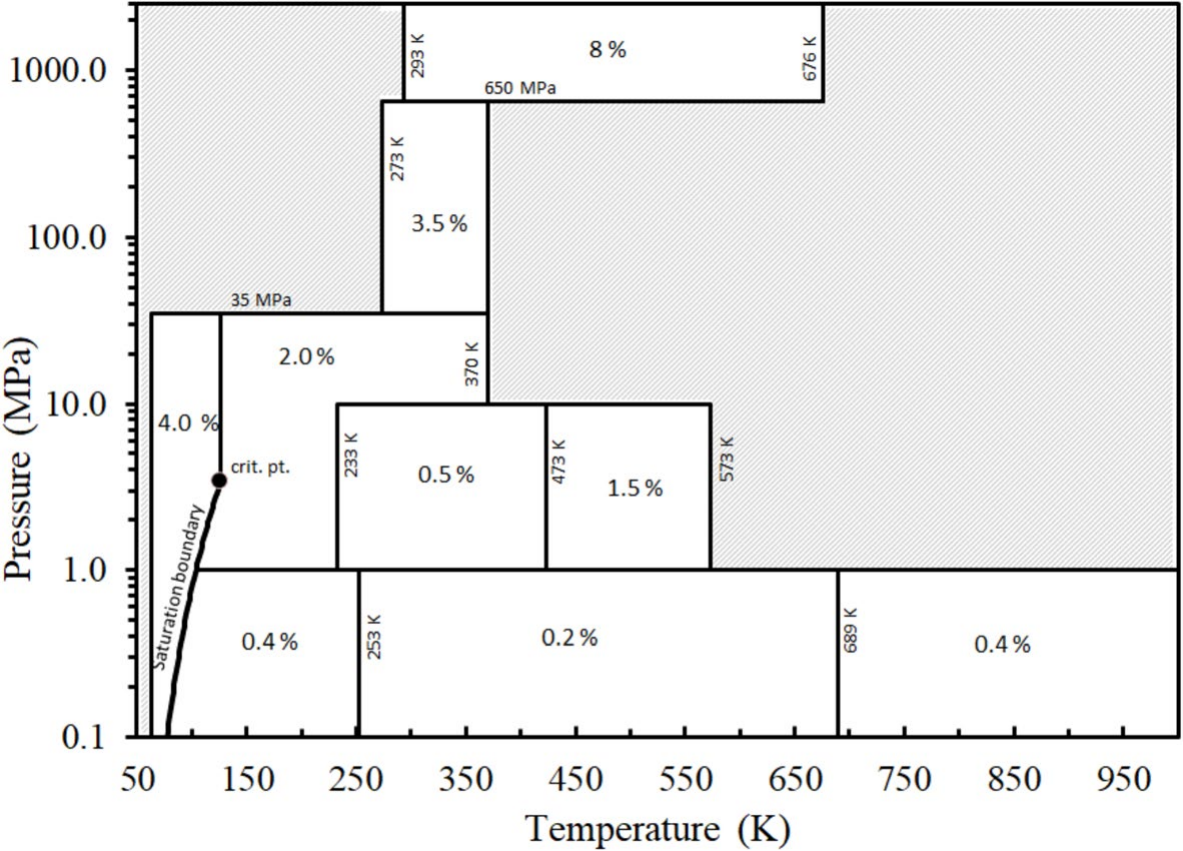
Estimated uncertainties for Eqs. 1,3,5

The present work is designed to be used with the equation of state for nitrogen of Span et al. [24], which has a domain of validity from the triple-point temperature of 63.151 to 1000 K and pressures up to 2200 MPa. The estimated uncertainty in viscosity, at *k* = 2 level, is based here primarily on comparisons with the most accurate experimental data in specific regions of the phase diagram as summarized in Fig. 14. For the low-pressure gas phase (*P* < 1 MPa), as discussed in Sect. 2.1, we adopted the dilute-gas limit viscosity formulation of Xiao et al. [11–13]. According to Xiao et al. [11–13], this formulation is recommended for the temperature range of 70 K to 3000 K with an estimated uncertainty of 0.4 % (at *k* = 2) over the entire range specified. Comparisons with the data sets of Vogel [32] and Humberg et al. [29] in Table 5 indicate that for the low-pressure gas (*P* < 1 MPa) the correlation has an estimated uncertainty of 0.2 % over the temperature range 253 K to 689 K. This is the same as the level of uncertainty of the experimental data. For temperatures above 689 K up to 3000 K, and for 70 K to 258 K, the uncertainty is as specified by Xiao et al., 0.4 %. Note that the minimum pressure shown is 0.1 MPa, but the correlation and the uncertainty discussed here goes down to the limit of zero. There are no gas-phase data below 70 K to validate the correlation down to the triple point, but the correlation behaves in a physically realistic manner and may be extrapolated to 30 K as discussed in Sec 2.2, but the uncertainty will be larger.

For the liquid phase (*T* < *T*_c_), there are validation data from near the triple-point temperature to the critical temperature, and the estimated uncertainty of the correlation is 4% for pressures up to 34 MPa. In the very high-pressure supercritical region, defined here as pressures from 650 to 10,700 MPa, the uncertainty estimate is based on comparisons with the data sets of Abramson [31, 38] and in this region the estimated uncertainty of the correlation is approximately 8 %, as indicated in Figs. 7 and 8. Note that at pressures above 2200 MPa the EOS must be extrapolated to provide densities and although the extrapolation behavior is smooth, the uncertainty in the density in this region is unknown. For the high-pressure region between 273 and 370 K from 35 to 650 MPa, the estimated uncertainty is 3.5 % based on comparisons with the data of Lazar and Vodar [69] and Vermesse [59]. For the moderate-pressure region, with pressures between 1 and 35 MPa, the uncertainty varies between 0.5 % and 2.0 %. The shaded areas in Fig. 14 indicate that there is a lack of sufficient high-accuracy experimental data to be able to perform comparisons to obtain an uncertainty estimate. Although data coverage is quite good for nitrogen, there still are areas at high temperatures and pressures that do not yet have adequate experimental data. Nevertheless, the correlation is expected to extrapolate in a physically realistic way and can be used in the shaded areas.

### Values for Computer Verification

Tables 7 and 8 provide values to assist the user in computer-program verification. For Table 7, there is no critical enhancement term, and Δ*η*_c_ = 1. The viscosity calculations are based on the tabulated temperatures and densities; the pressure is calculated from the EOS of Span et al. [24] and is for information only.

**Table 7:** Viscosity values of nitrogen at selected temperatures and densities for computer verification

*P* (MPa)	*T* (K)	*ρ* (kg·m^−3^)	*η* (μPa·s)
0.000000	90	0	6.07115583
3.24893	90	756	108.42550781
0.000000	300	0	17.83446070
2.48533	300	28	18.23478803
95.2619	300	560	50.59605975

**Table 8:** Viscosity values of nitrogen at selected temperatures and densities for computer verification near the critical point (*T*_c_ = 126.192 K) [24]

*T* (K)	*Ρ* (kg·m^−3^)	*η*_0_ (μPa·s)	*η*_res_ (μPa·s)	*ξ* (nm)	Δ*η*_c_	*η* (μPa·s)
126.192	265	8.43716205	7.20032032	4.24379793	1.03344348	16.16045420
126.212	333	8.43843818	11.03805357	13.96074161	1.09122274	21.25319077
126.952	300	8.48562461	9.04720938	3.38864120	1.02554760	17.98075591

## Conclusions

A new wide-ranging correlation for the viscosity of nitrogen was developed based on critically evaluated experimental data and theoretical results. It is formulated as a function of temperature and density and is designed to be used with the equation of state of Span et al. [24]. The domain of validity of the equation of state and the new viscosity correlation is from the triple-point temperature up to 1000 K and pressures up to 2200 MPa. Based on comparisons with available viscosity data at high pressure, we feel it is safe to extrapolate the correlation to cover the range 294 K < *T* < 677 K at pressures up to 10.7 GPa. The correlation also may be used for the dilute gas at temperatures up to 3000 K.

The new viscosity correlation offers improvement over the correlation of Lemmon and Jacobsen [7] that currently is used for reference calculations. There are two main areas of improvement: (1) the low-pressure gas region (*P* < 1 MPa), and (2) for pressures above 650 MPa. The new correlation reproduces the reference value of the viscosity of nitrogen at 25 °C and zero density of 17.7494 μPa·s proposed by Berg and Moldover [14]. In addition, for the low-pressure gas region, the estimated uncertainty of the present work is 0.2 % over the temperature range 253 K to 689 K, and 0.4 % for 70 K < *T* < 253 K and for 689 K < *T* < 3000 K. The Lemmon and Jacobsen correlation [7] has an estimated uncertainty of 0.5 % in the low-pressure gas region and does not reproduce the reference point at 25 °C. The improvement is due to the inclusion of the dilute-gas correlation proposed by Xiao et al. [11–13] that incorporates developments in theory of the dilute gas and will be useful for the calibration of gas flow meters. For very high pressures (from 650 to 2200 MPa), the Lemmon and Jacobsen correlation shows deviations from experimental data that exceed 25 %, while the new correlation does not exceed 10 %. Outside of these two regions, the results of the two models are very similar. Finally, care has been taken to control the extrapolation behavior to allow the use of the correlation in applications such as corresponding-states models and some mixture models that require computation at conditions outside of the physical limitations of pure nitrogen (such as temperatures down to 30 K and state points that would correspond to the 2-phase region of pure nitrogen).

## Supplementary Information

Below is the link to the electronic supplementary material.

Supplementary file1 (TXT 52 KB) This file should be renamed NITROGEN.FLD for use with REFPROP

Supplementary file2 (PDF 3445 KB)

## Data Availability

No datasets were generated or analysed during the current study.
