# Supplementary material for: Reference Correlation for the Viscosity of Nitrogen from the Triple Point to 1000 K and Pressures up to 2200 MPa
Source: Int J Thermophys. 2024 Oct 10;45(10):146. doi: 10.1007/s10765-024-03440-1 (PMC11466908; doi:10.1007/s10765-024-03440-1)
Supplement: Supplementary file 2 — Supplementary file2 (PDF 3445 KB) [file 10765_2024_3440_MOESM2_ESM.pdf]

Supplementary Information

1. Additional comparisons of the present correlation, Eq. 1,3,5 with the primary data in the pressure range 1 MPa < p < 100 MPa

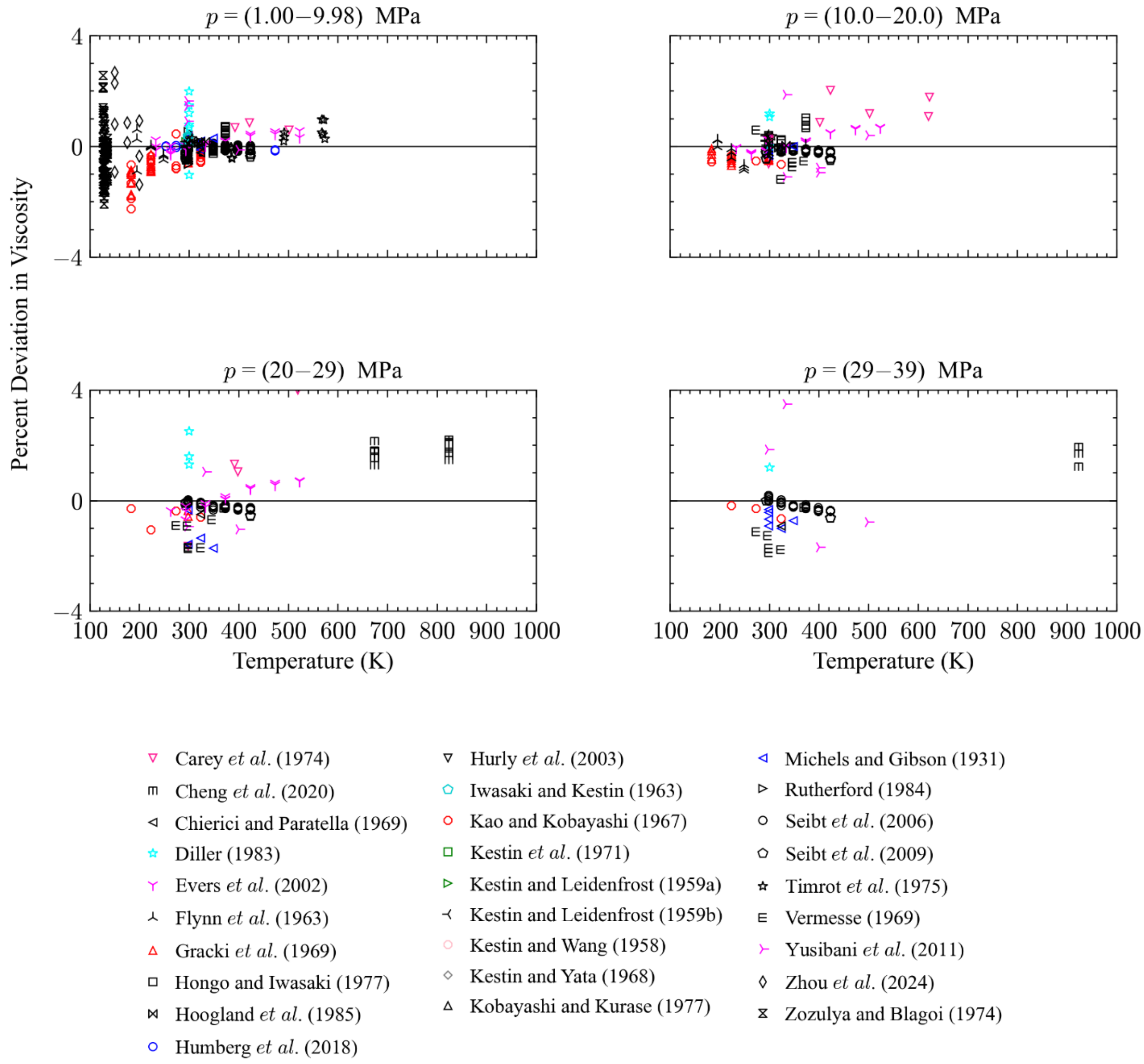

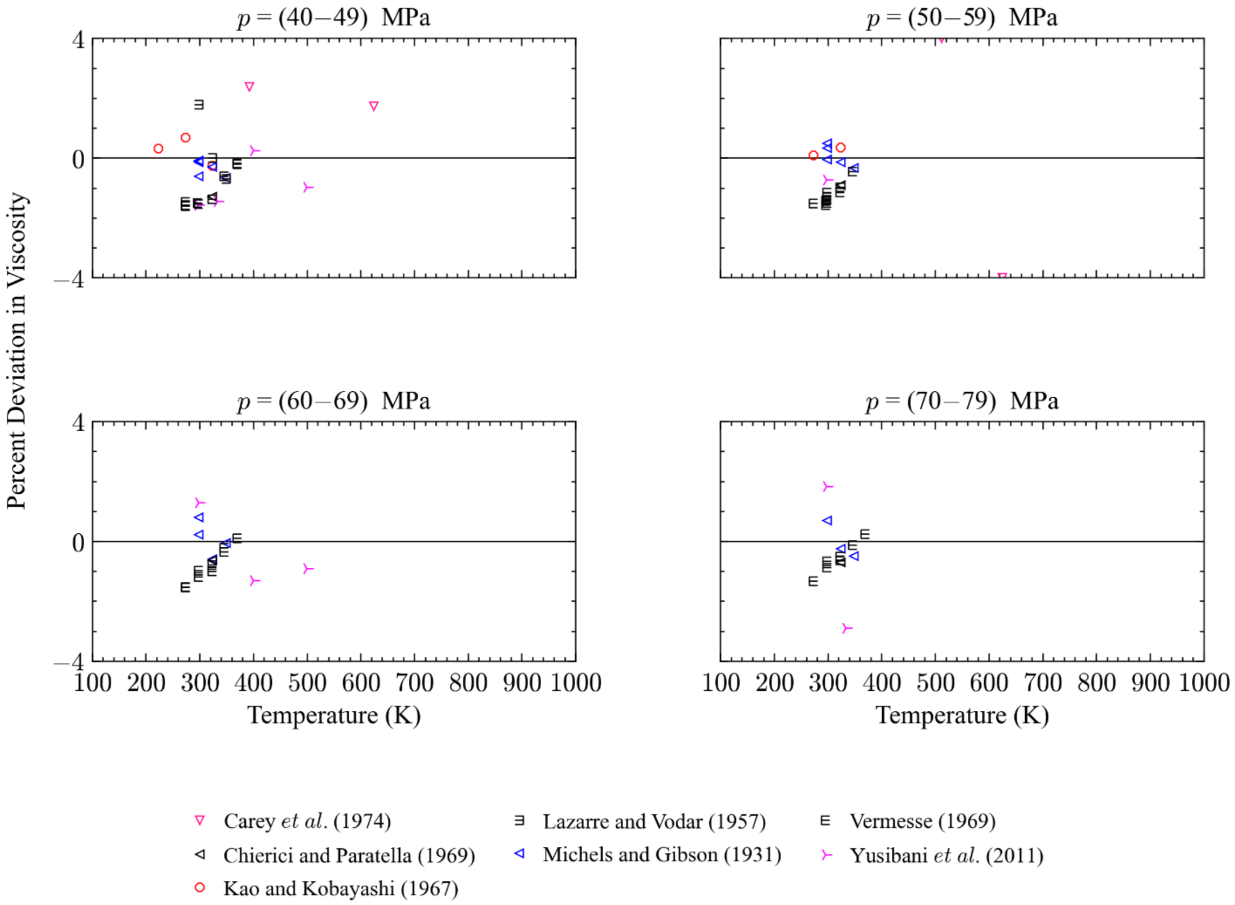
p = (40–49) MPa
p = (50–59) MPa
p = (60–69) MPa
p = (70–79) MPa
Percent Deviation in Viscosity
Temperature (K)
Carey et al. (1974)
Chierici and Paratella (1969)
Kao and Kobayashi (1967)
Lazarre and Vodar (1957)
Michels and Gibson (1931)
Vermesse (1969)
Yusibani et al. (2011)

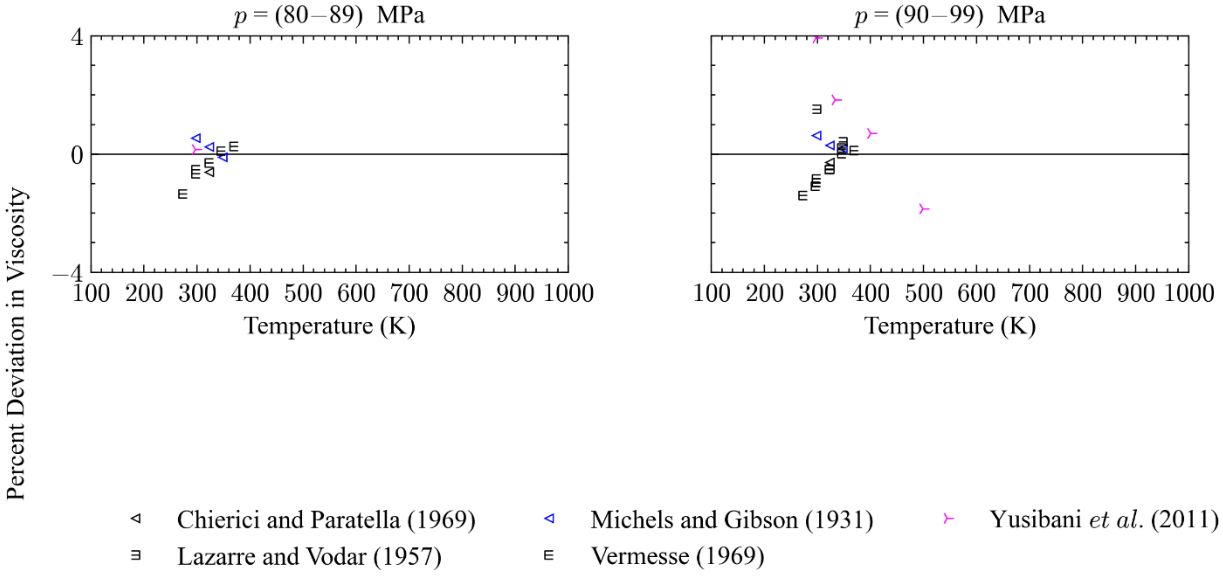
p = (80–89) MPa
p = (90–99) MPa
Percent Deviation in Viscosity
Temperature (K)
Chierici and Paratella (1969)
Lazarre and Vodar (1957)
Michels and Gibson (1931)
Vermesse (1969)
Yusibani et al. (2011)

2. Additional comparisons of the present correlation, Eq. 1,3,5 with the primary data for selected nominal temperatures

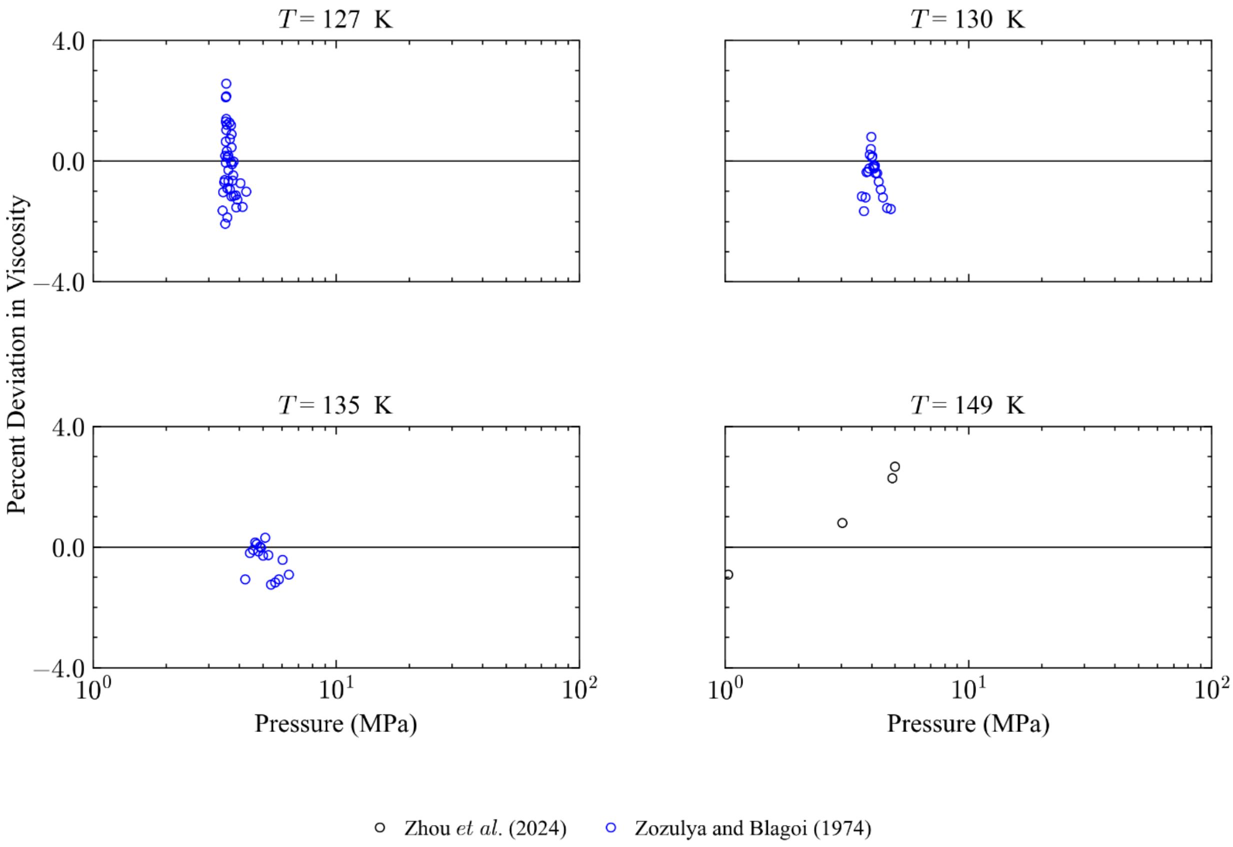

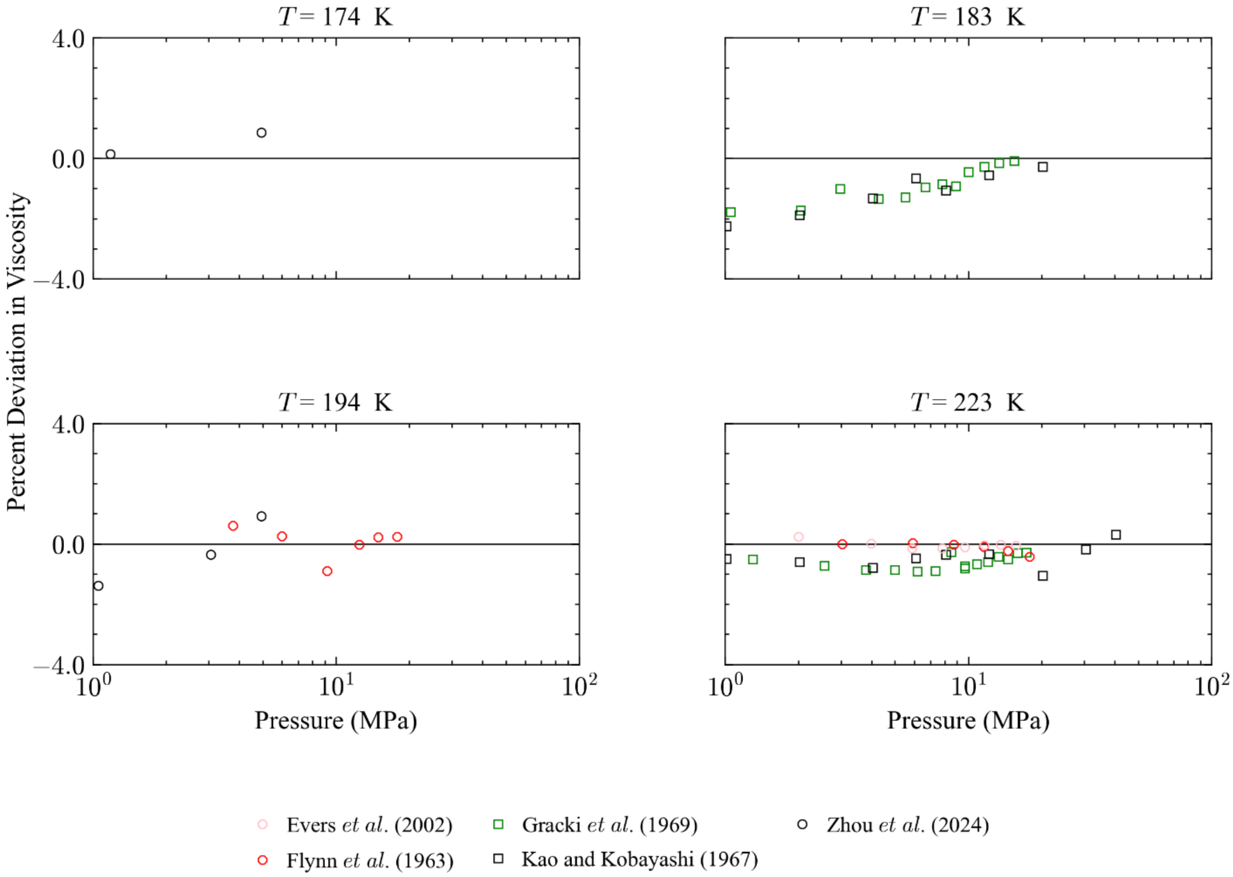
T = 174 K
T = 183 K
T = 194 K
T = 223 K
Percent Deviation in Viscosity
4.0
0.0
−4.0
10^0
10^1
10^2
Pressure (MPa)
Evers et al. (2002)
Flynn et al. (1963)
Gracki et al. (1969)
Kao and Kobayashi (1967)
Zhou et al. (2024)

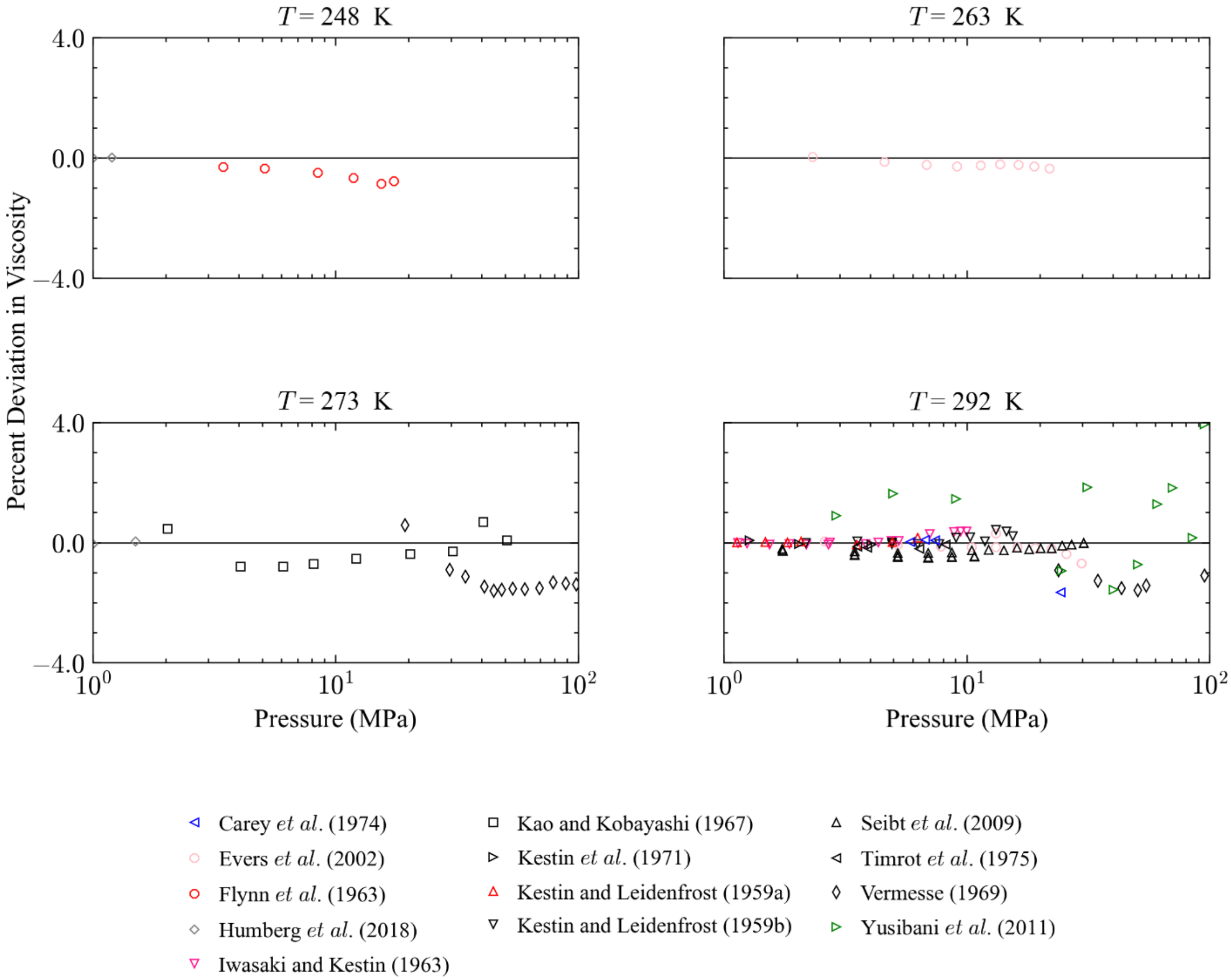

T = 248 K
T = 263 K
T = 273 K
T = 292 K
4.0
0.0
−4.0
Percent Deviation in Viscosity
10^0
10^1
10^2
Pressure (MPa)
Carey et al. (1974)
Evers et al. (2002)
Flynn et al. (1963)
Humberg et al. (2018)
Iwasaki and Kestin (1963)
Kao and Kobayashi (1967)
Kestin et al. (1971)
Kestin and Leidenfrost (1959a)
Kestin and Leidenfrost (1959b)
Seibt et al. (2009)
Timrot et al. (1975)
Vermesse (1969)
Yusibani et al. (2011)

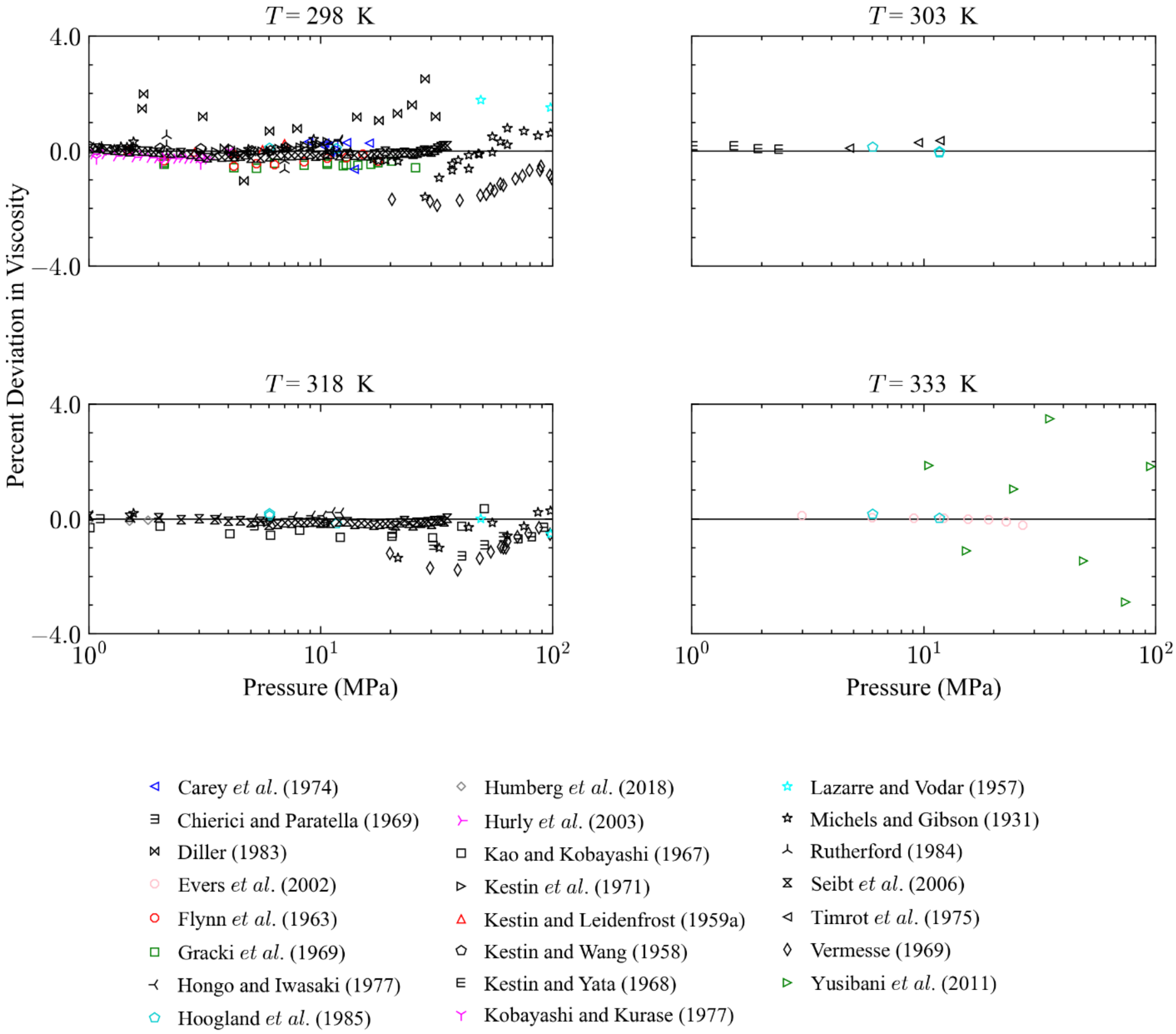

T = 298 K
T = 303 K
T = 318 K
T = 333 K
Percent Deviation in Viscosity
4.0
0.0
−4.0
10^0
10^1
10^2
Pressure (MPa)
Carey et al. (1974)
Chierici and Paratella (1969)
Diller (1983)
Evers et al. (2002)
Flynn et al. (1963)
Gracki et al. (1969)
Hongo and Iwasaki (1977)
Hoogland et al. (1985)
Humberg et al. (2018)
Hurly et al. (2003)
Kao and Kobayashi (1967)
Kestin et al. (1971)
Kestin and Leidenfrost (1959a)
Kestin and Wang (1958)
Kestin and Yata (1968)
Kobayashi and Kurase (1977)
Lazarre and Vodar (1957)
Michels and Gibson (1931)
Rutherford (1984)
Seibt et al. (2006)
Timrot et al. (1975)
Vermesse (1969)
Yusibani et al. (2011)

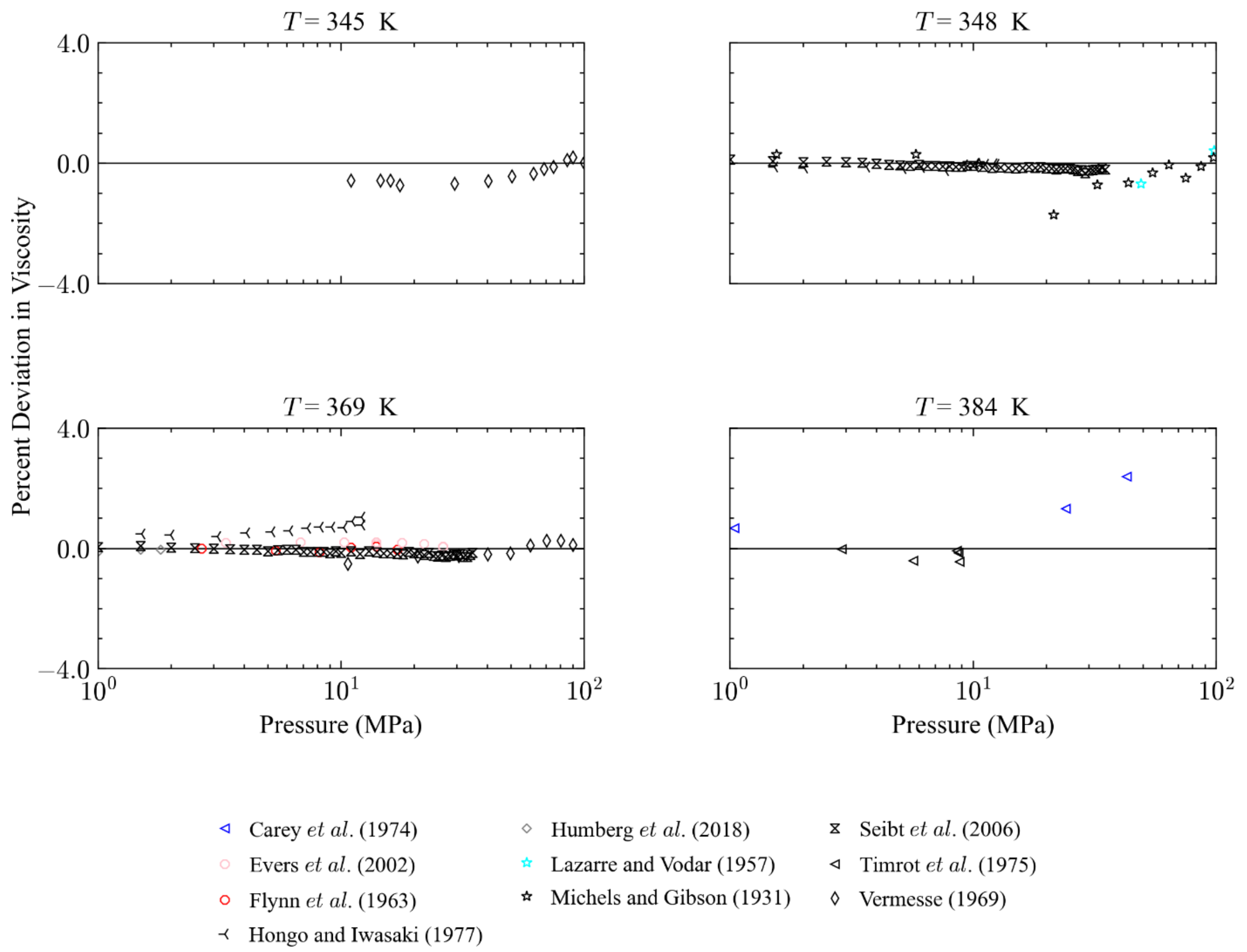

T = 345 K
T = 348 K
T = 369 K
T = 384 K
4.0
0.0
−4.0
Percent Deviation in Viscosity
10^0
10^1
10^2
Pressure (MPa)
Carey et al. (1974)
Evers et al. (2002)
Flynn et al. (1963)
Hongo and Iwasaki (1977)
Humberg et al. (2018)
Lazarre and Vodar (1957)
Michels and Gibson (1931)
Seibt et al. (2006)
Timrot et al. (1975)
Vermesse (1969)

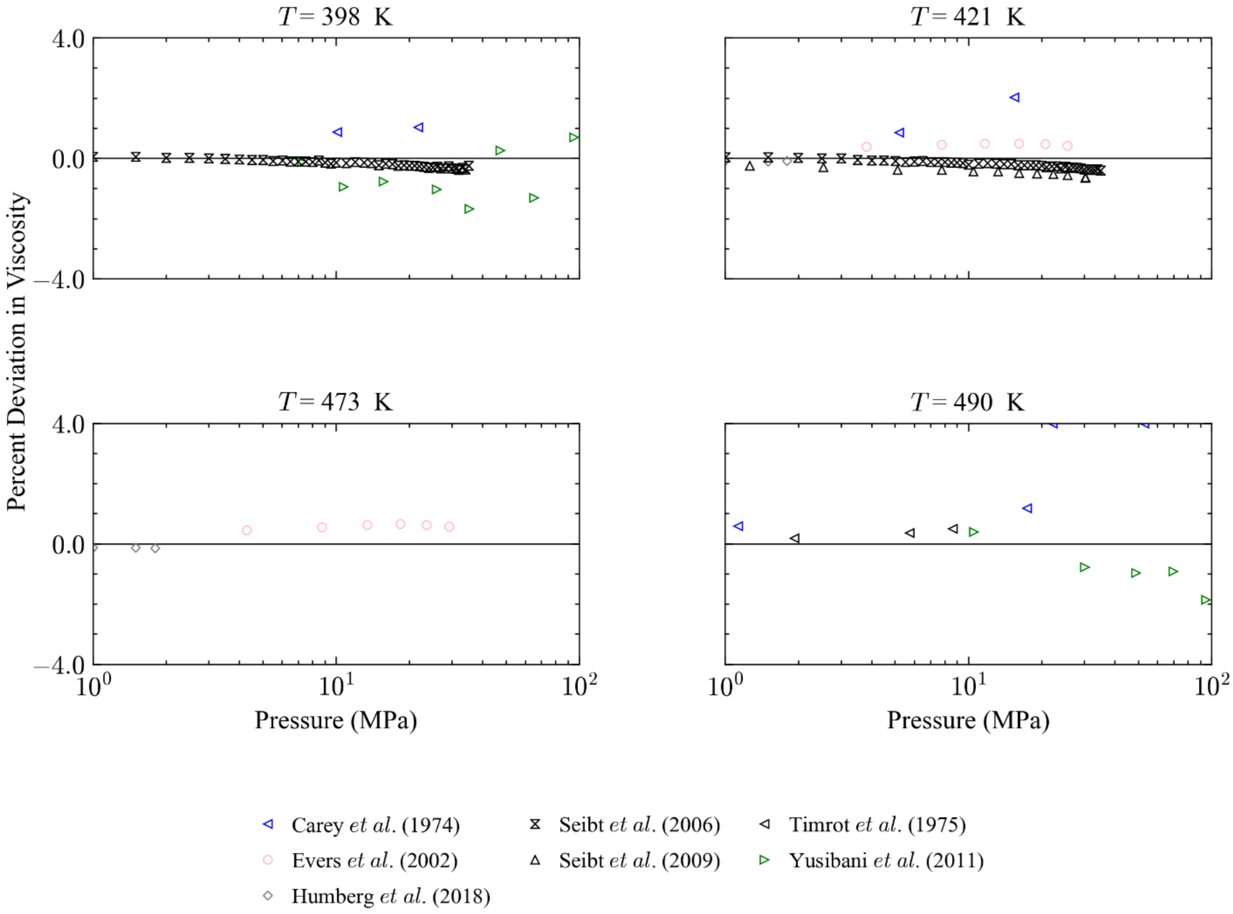
$T = 398$ K
$T = 421$ K
$T = 473$ K
$T = 490$ K
4.0
0.0
−4.0
Percent Deviation in Viscosity
10^0
10^1
10^2
Pressure (MPa)
Carey *et al.* (1974)
Evers *et al.* (2002)
Humberg *et al.* (2018)
Seibt *et al.* (2006)
Seibt *et al.* (2009)
Timrot *et al.* (1975)
Yusibani *et al.* (2011)

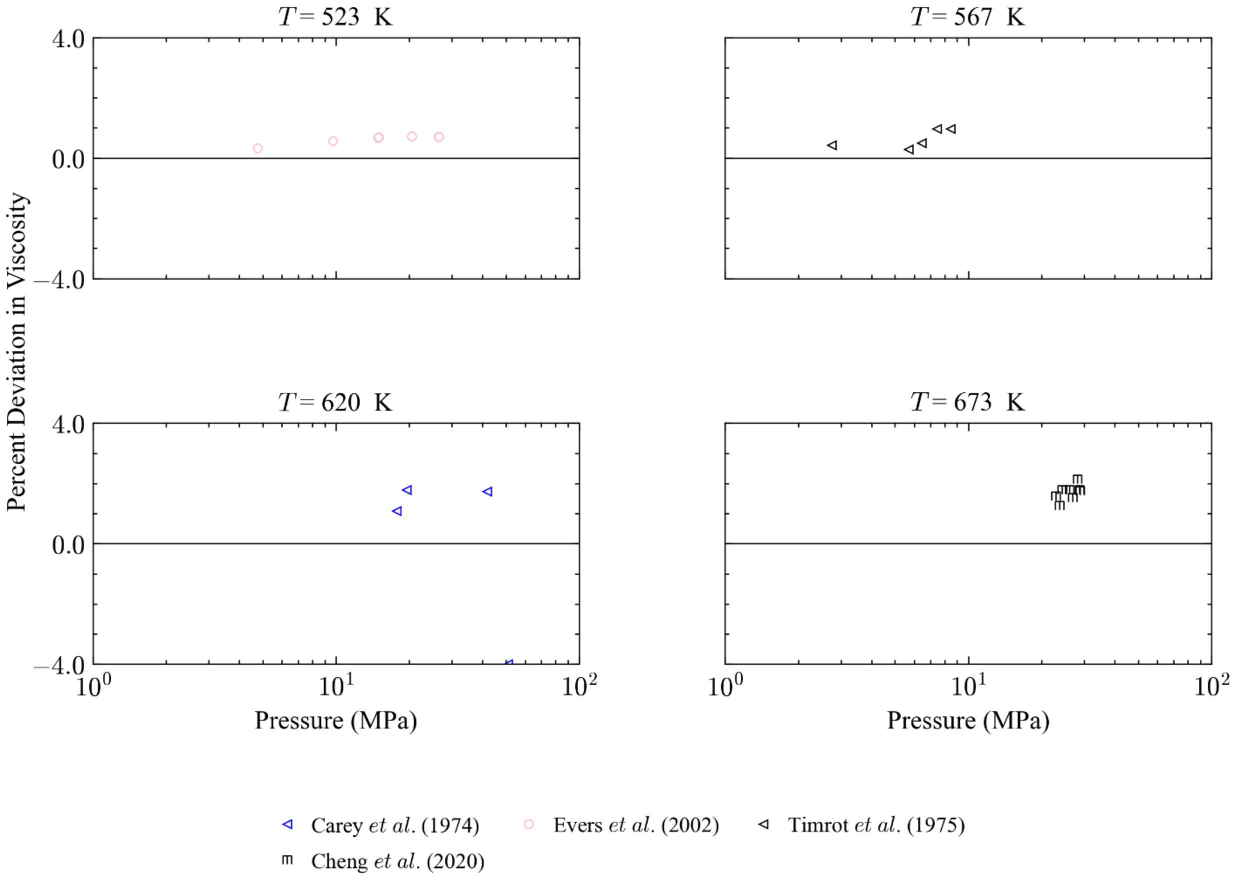

T = 523 K
T = 567 K
T = 620 K
T = 673 K
Percent Deviation in Viscosity
4.0
0.0
−4.0
Pressure (MPa)
Carey et al. (1974)
Evers et al. (2002)
Timrot et al. (1975)
Cheng et al. (2020)

3. Additional comparisons of the present correlation, Eq. 1,3,5 with secondary data

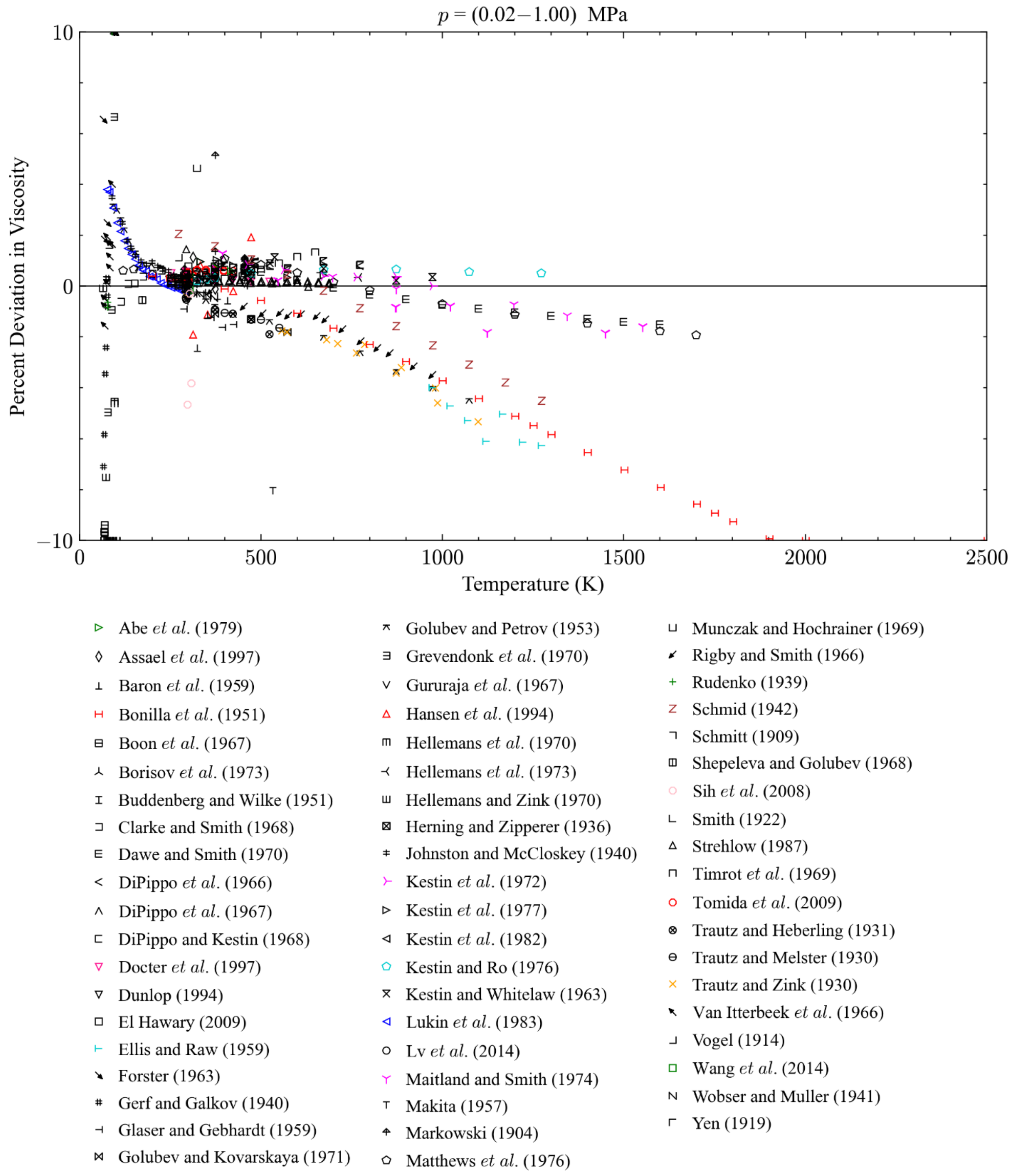

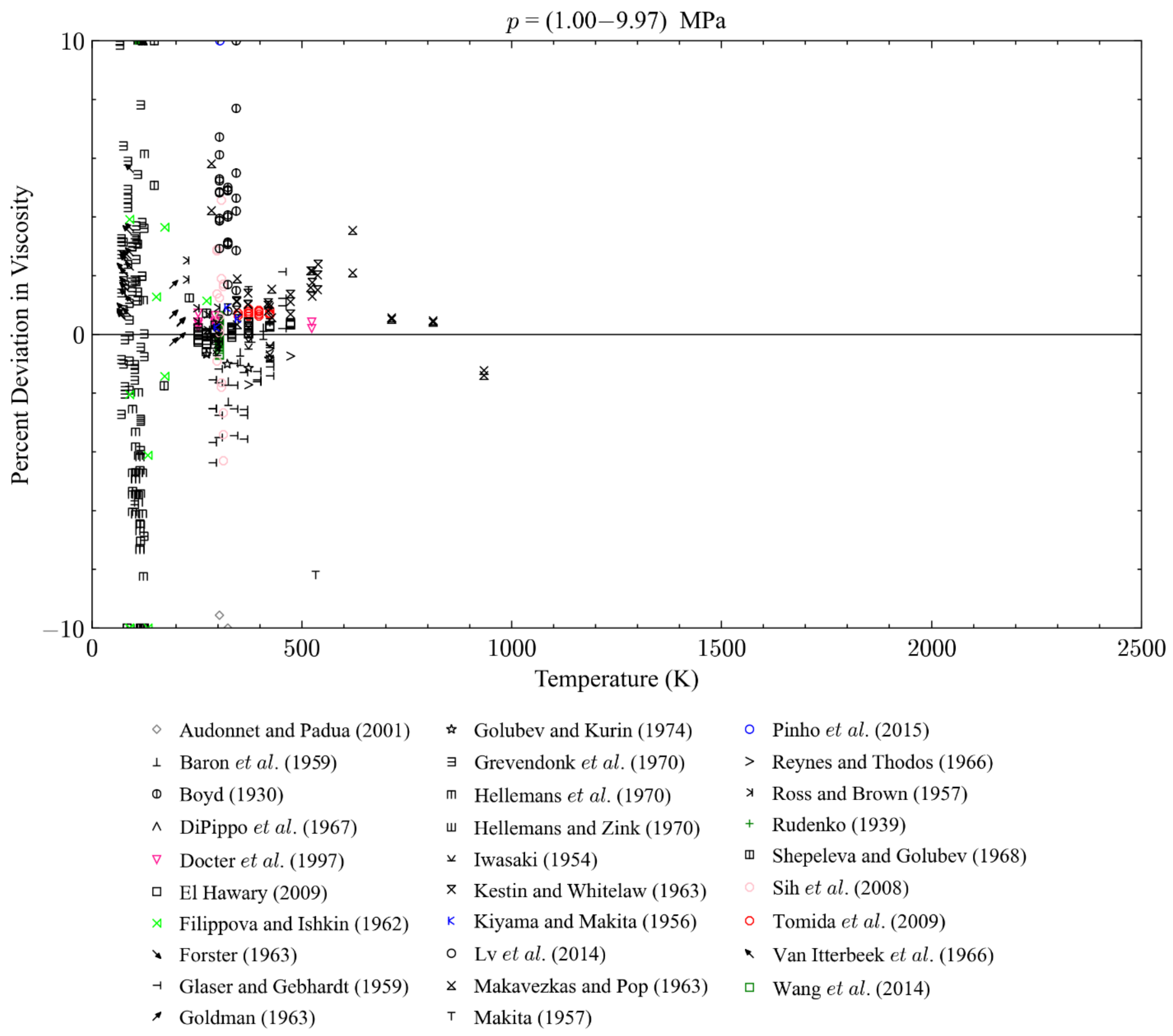

p = (1.00–9.97) MPa
Percent Deviation in Viscosity
Temperature (K)
10
0
–10
0
500
1000
1500
2000
2500
Audonnet and Padua (2001)
Baron et al. (1959)
Boyd (1930)
DiPippo et al. (1967)
Docter et al. (1997)
El Hawary (2009)
Filippova and Ishkin (1962)
Forster (1963)
Glaser and Gebhardt (1959)
Goldman (1963)
Golubev and Kurin (1974)
Grevendonk et al. (1970)
Hellemans et al. (1970)
Hellemans and Zink (1970)
Iwasaki (1954)
Kestin and Whitelaw (1963)
Kiyama and Makita (1956)
Lv et al. (2014)
Makavezkas and Pop (1963)
Makita (1957)
Pinho et al. (2015)
Reynes and Thodos (1966)
Ross and Brown (1957)
Rudenko (1939)
Shepeleva and Golubev (1968)
Sih et al. (2008)
Tomida et al. (2009)
Van Itterbeek et al. (1966)
Wang et al. (2014)

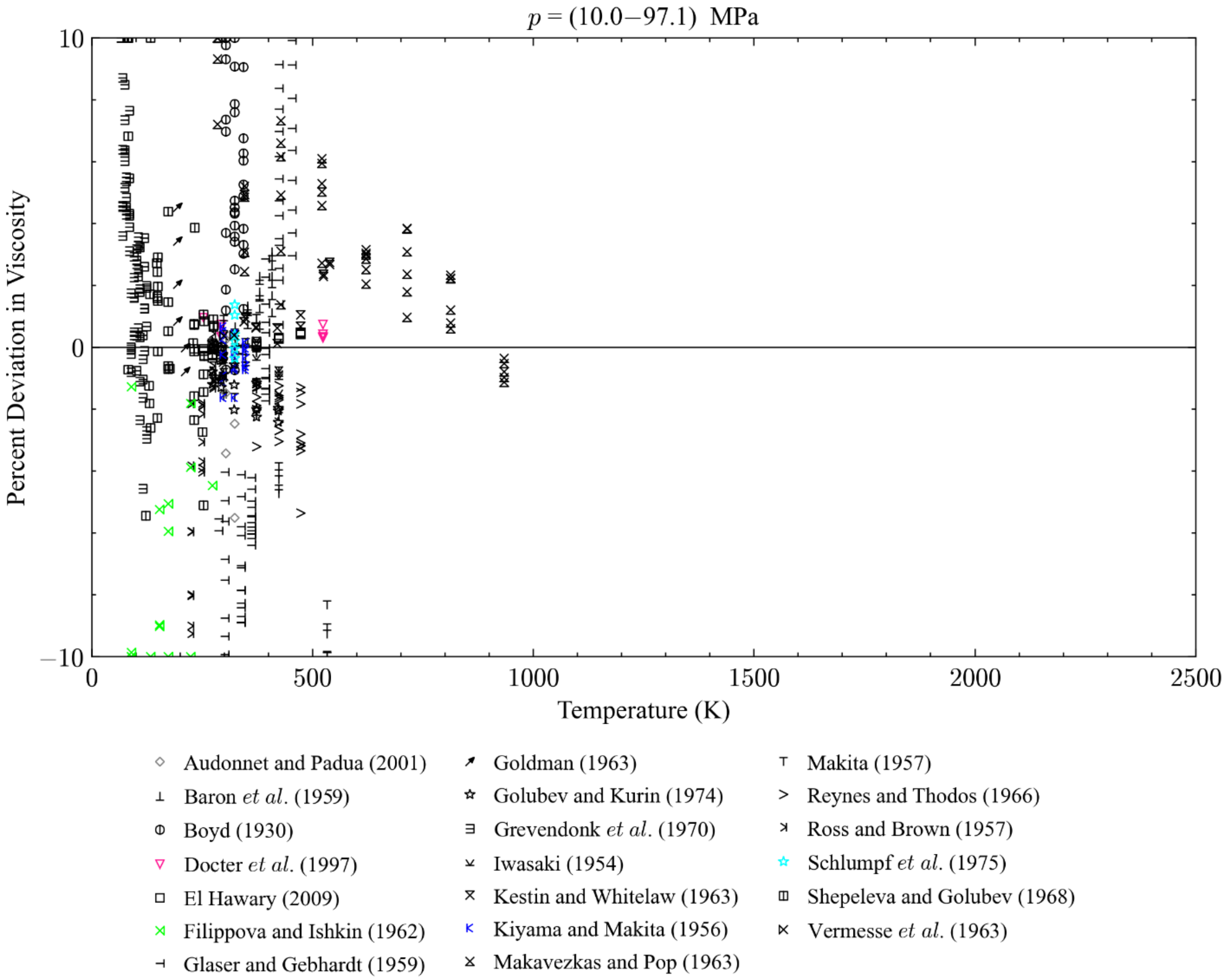

p = (10.0−97.1) MPa
Percent Deviation in Viscosity
Temperature (K)
10
0
−10
0
500
1000
1500
2000
2500
Audonnet and Padua (2001)
Baron et al. (1959)
Boyd (1930)
Docter et al. (1997)
El Hawary (2009)
Filippova and Ishkin (1962)
Glaser and Gebhardt (1959)
Goldman (1963)
Golubev and Kurin (1974)
Grevendonk et al. (1970)
Iwasaki (1954)
Kestin and Whitelaw (1963)
Kiyama and Makita (1956)
Makavezkas and Pop (1963)
Makita (1957)
Reynes and Thodos (1966)
Ross and Brown (1957)
Schlumpf et al. (1975)
Shepeleva and Golubev (1968)
Vermesse et al. (1963)

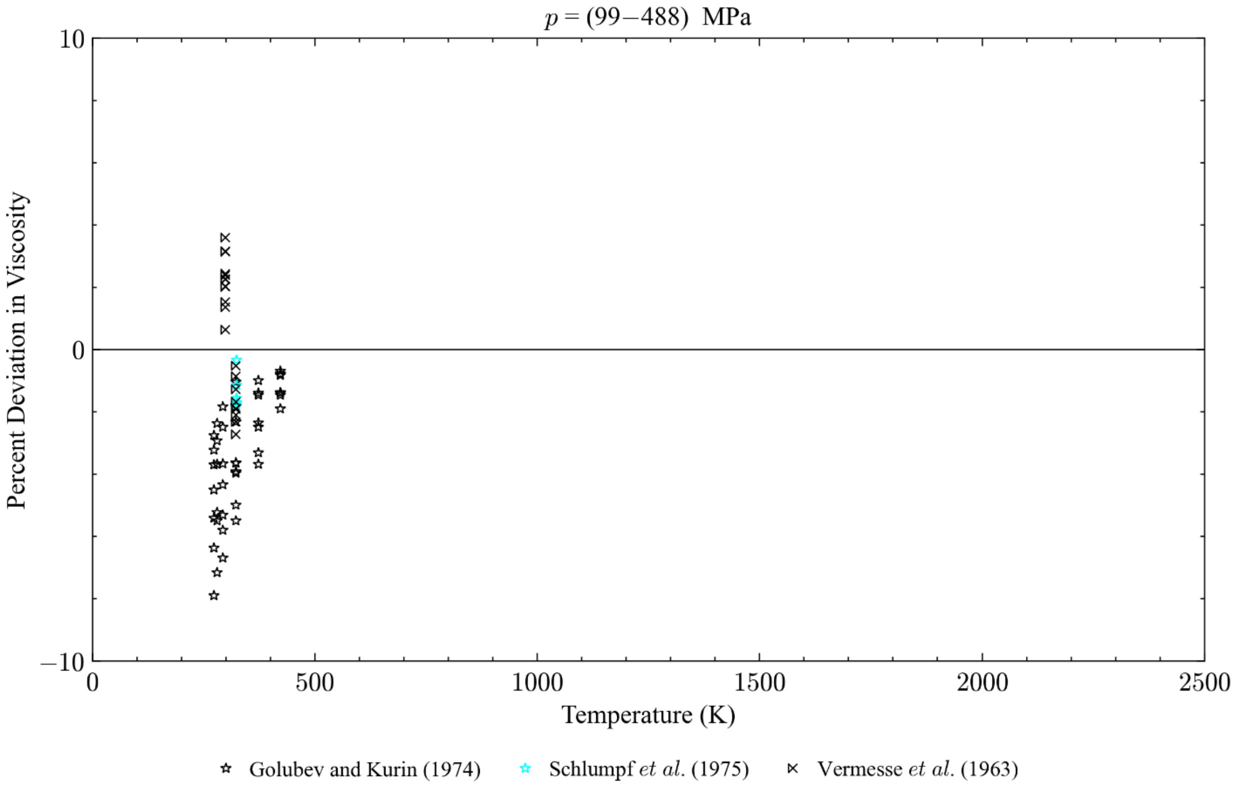

p = (99−488) MPa
Percent Deviation in Viscosity
10
0
−10
0
500
1000
1500
2000
2500
Temperature (K)
Golubev and Kurin (1974)
Schlumpf et al. (1975)
Vermesse et al. (1963)

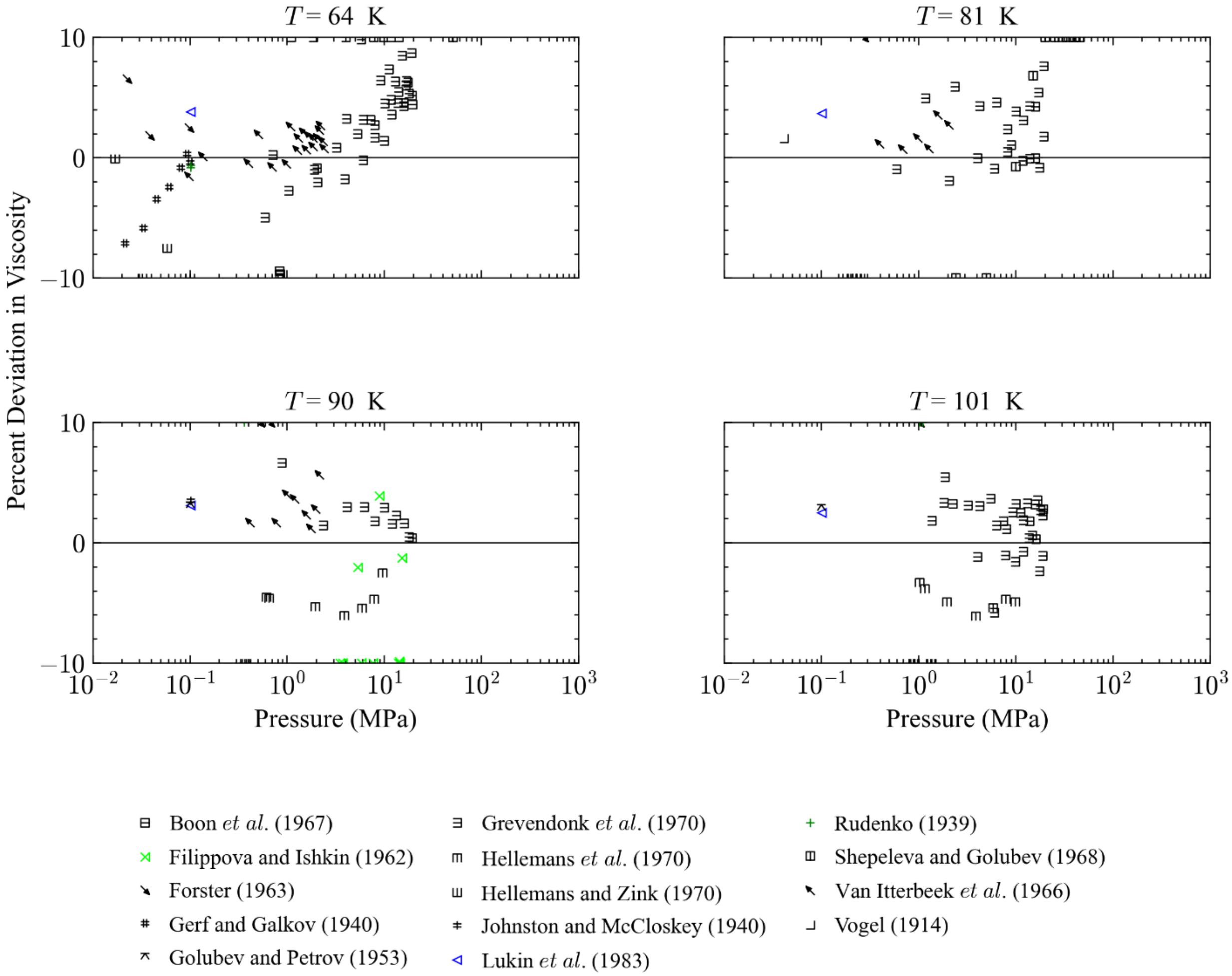
T = 64 K
T = 81 K
T = 90 K
T = 101 K
Percent Deviation in Viscosity
Pressure (MPa)
Boon et al. (1967)
Filippova and Ishkin (1962)
Forster (1963)
Gerf and Galkov (1940)
Golubev and Petrov (1953)
Grevendonk et al. (1970)
Hellemans et al. (1970)
Hellemans and Zink (1970)
Johnston and McCloskey (1940)
Lukin et al. (1983)
Rudenko (1939)
Shepeleva and Golubev (1968)
Van Itterbeek et al. (1966)
Vogel (1914)

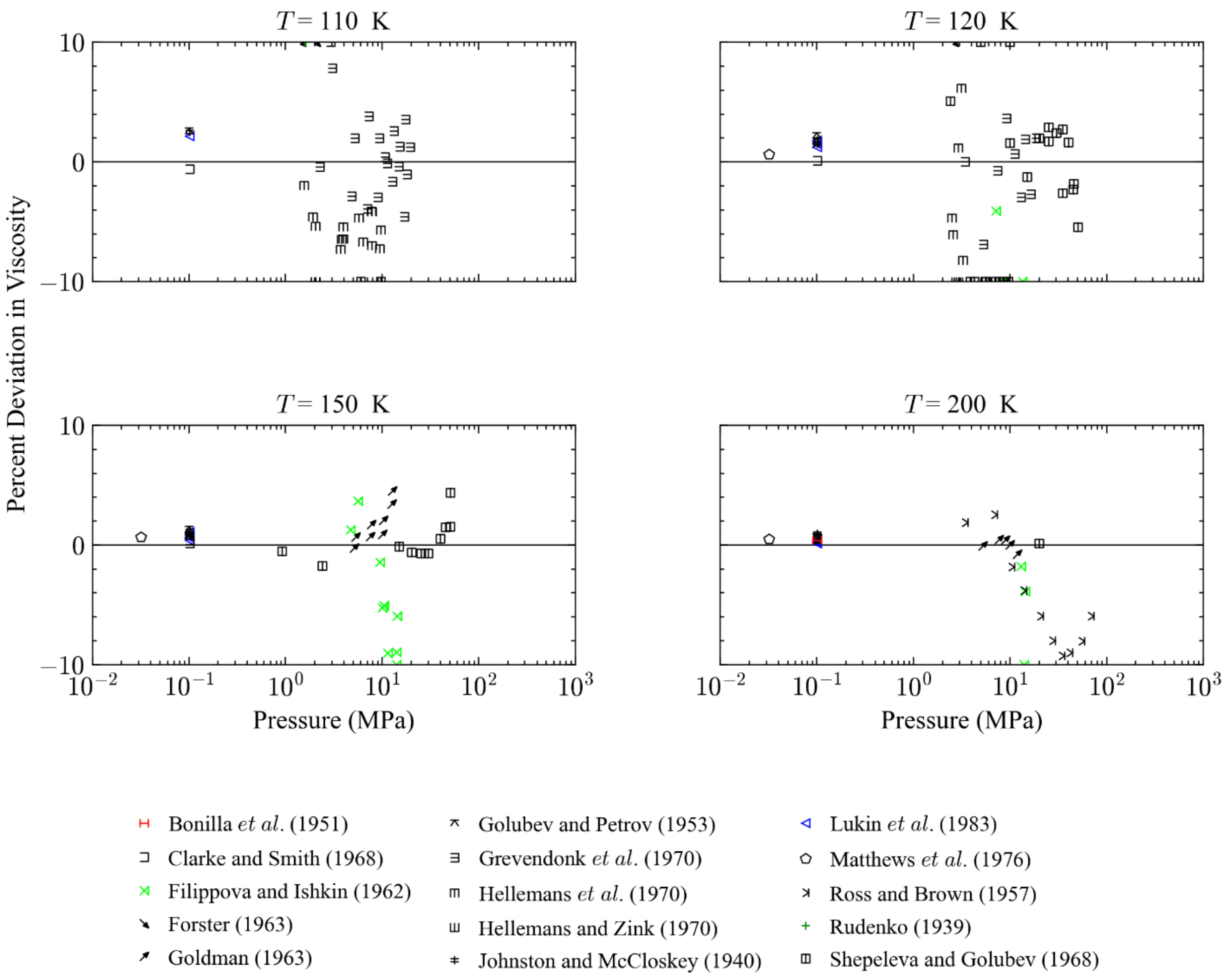

T = 110 K
T = 120 K
T = 150 K
T = 200 K
Percent Deviation in Viscosity
Pressure (MPa)
Pressure (MPa)
10
0
−10
10^{-2}
10^{-1}
10^{0}
10^{1}
10^{2}
10^{3}
Bonilla et al. (1951)
Clarke and Smith (1968)
Filippova and Ishkin (1962)
Forster (1963)
Goldman (1963)
Golubev and Petrov (1953)
Grevendonk et al. (1970)
Hellemans et al. (1970)
Hellemans and Zink (1970)
Johnston and McCloskey (1940)
Lukin et al. (1983)
Matthews et al. (1976)
Ross and Brown (1957)
Rudenko (1939)
Shepeleva and Golubev (1968)

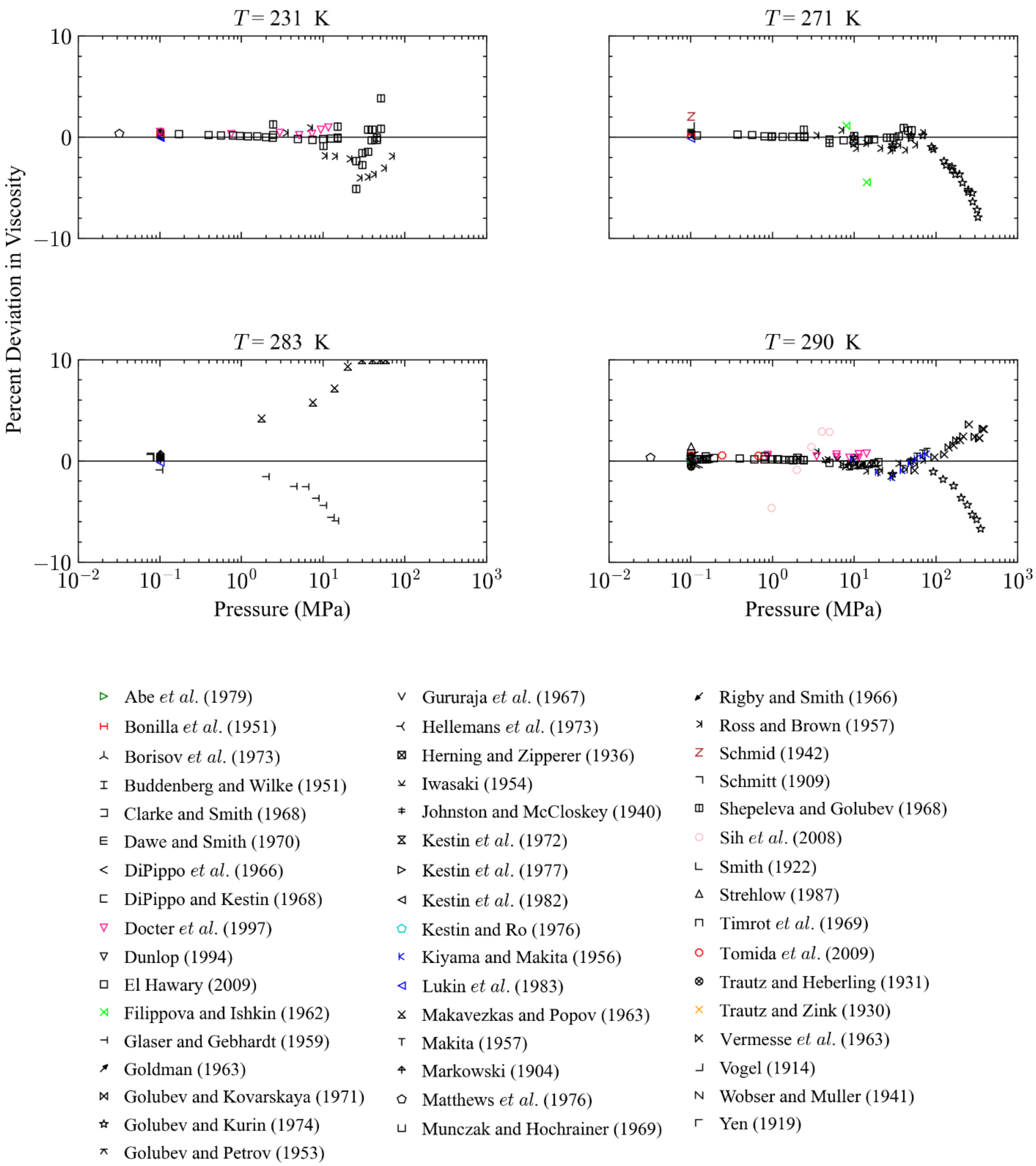
T = 231 K
T = 271 K
T = 283 K
T = 290 K
Percent Deviation in Viscosity
10
0
−10
10^{-2} 10^{-1} 10^{0} 10^{1} 10^{2} 10^{3}
Pressure (MPa)
Abe et al. (1979)
Bonilla et al. (1951)
Borisov et al. (1973)
Buddenberg and Wilke (1951)
Clarke and Smith (1968)
Dawe and Smith (1970)
DiPippo et al. (1966)
DiPippo and Kestin (1968)
Docter et al. (1997)
Dunlop (1994)
El Hawary (2009)
Filippova and Ishkin (1962)
Glaser and Gebhardt (1959)
Goldman (1963)
Golubev and Kovarskaya (1971)
Golubev and Kurin (1974)
Golubev and Petrov (1953)
Gururaja et al. (1967)
Hellemans et al. (1973)
Herning and Zipperer (1936)
Iwasaki (1954)
Johnston and McCloskey (1940)
Kestin et al. (1972)
Kestin et al. (1977)
Kestin et al. (1982)
Kestin and Ro (1976)
Kiyama and Makita (1956)
Lukin et al. (1983)
Makavezkas and Popov (1963)
Makita (1957)
Markowski (1904)
Matthews et al. (1976)
Munczak and Hochrainer (1969)
Rigby and Smith (1966)
Ross and Brown (1957)
Schmid (1942)
Schmitt (1909)
Shepeleva and Golubev (1968)
Sih et al. (2008)
Smith (1922)
Strehlow (1987)
Timrot et al. (1969)
Tomida et al. (2009)
Trautz and Heberling (1931)
Trautz and Zink (1930)
Vermesse et al. (1963)
Vogel (1914)
Wobser and Muller (1941)
Yen (1919)

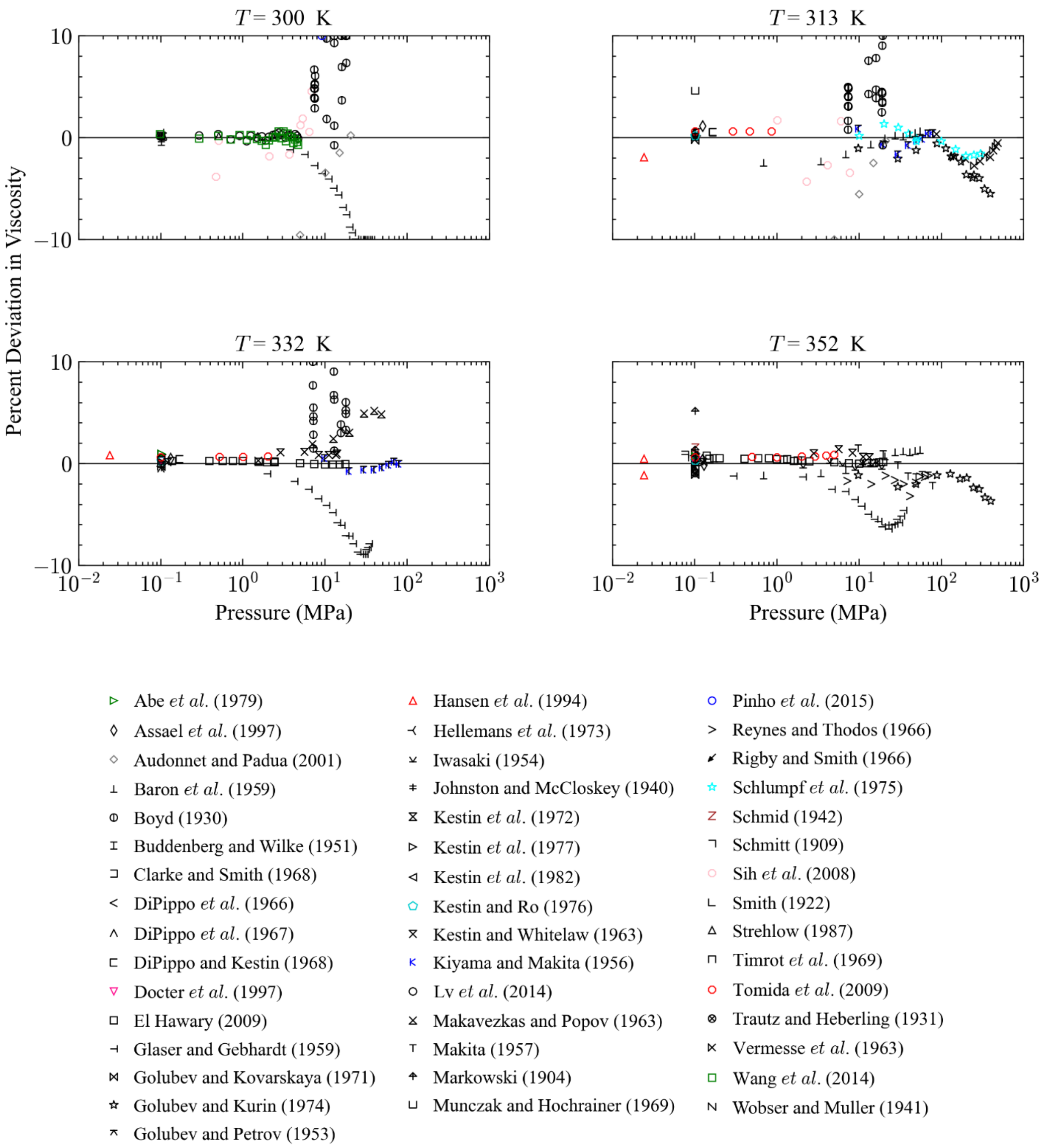

T = 300 K
T = 313 K
T = 332 K
T = 352 K
Percent Deviation in Viscosity
Pressure (MPa)
Abe et al. (1979)
Assael et al. (1997)
Audonnet and Padua (2001)
Baron et al. (1959)
Boyd (1930)
Buddenberg and Wilke (1951)
Clarke and Smith (1968)
DiPippo et al. (1966)
DiPippo et al. (1967)
DiPippo and Kestin (1968)
Docter et al. (1997)
El Hawary (2009)
Glaser and Gebhardt (1959)
Golubev and Kovarskaya (1971)
Golubev and Kurin (1974)
Golubev and Petrov (1953)
Hansen et al. (1994)
Hellemans et al. (1973)
Iwasaki (1954)
Johnston and McCloskey (1940)
Kestin et al. (1972)
Kestin et al. (1977)
Kestin et al. (1982)
Kestin and Ro (1976)
Kestin and Whitelaw (1963)
Kiyama and Makita (1956)
Lv et al. (2014)
Makavezkas and Popov (1963)
Makita (1957)
Markowski (1904)
Munczak and Hochrainer (1969)
Pinho et al. (2015)
Reynes and Thodos (1966)
Rigby and Smith (1966)
Schlumpf et al. (1975)
Schmid (1942)
Schmitt (1909)
Sih et al. (2008)
Smith (1922)
Strehlow (1987)
Timrot et al. (1969)
Tomida et al. (2009)
Trautz and Heberling (1931)
Vermesse et al. (1963)
Wang et al. (2014)
Wobser and Muller (1941)

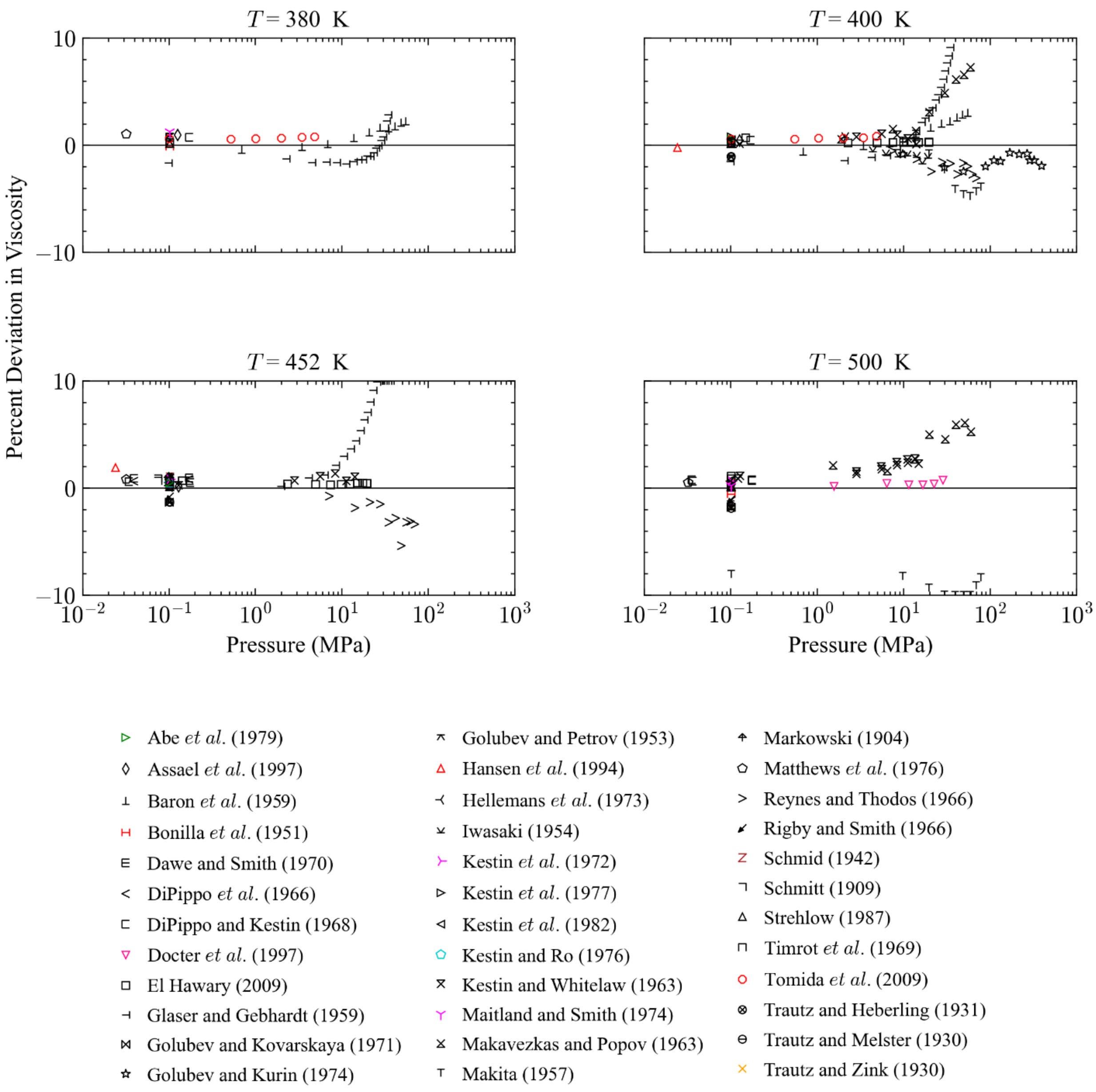

T = 380 K
T = 400 K
T = 452 K
T = 500 K
Percent Deviation in Viscosity
Pressure (MPa)
Abe et al. (1979)
Assael et al. (1997)
Baron et al. (1959)
Bonilla et al. (1951)
Dawe and Smith (1970)
DiPippo et al. (1966)
DiPippo and Kestin (1968)
Docter et al. (1997)
El Hawary (2009)
Glaser and Gebhardt (1959)
Golubev and Kovarskaya (1971)
Golubev and Kurin (1974)
Golubev and Petrov (1953)
Hansen et al. (1994)
Hellemans et al. (1973)
Iwasaki (1954)
Kestin et al. (1972)
Kestin et al. (1977)
Kestin et al. (1982)
Kestin and Ro (1976)
Kestin and Whitelaw (1963)
Maitland and Smith (1974)
Makavezkas and Popov (1963)
Makita (1957)
Markowski (1904)
Matthews et al. (1976)
Reynes and Thodos (1966)
Rigby and Smith (1966)
Schmid (1942)
Schmitt (1909)
Strehlow (1987)
Timrot et al. (1969)
Tomida et al. (2009)
Trautz and Heberling (1931)
Trautz and Melster (1930)
Trautz and Zink (1930)

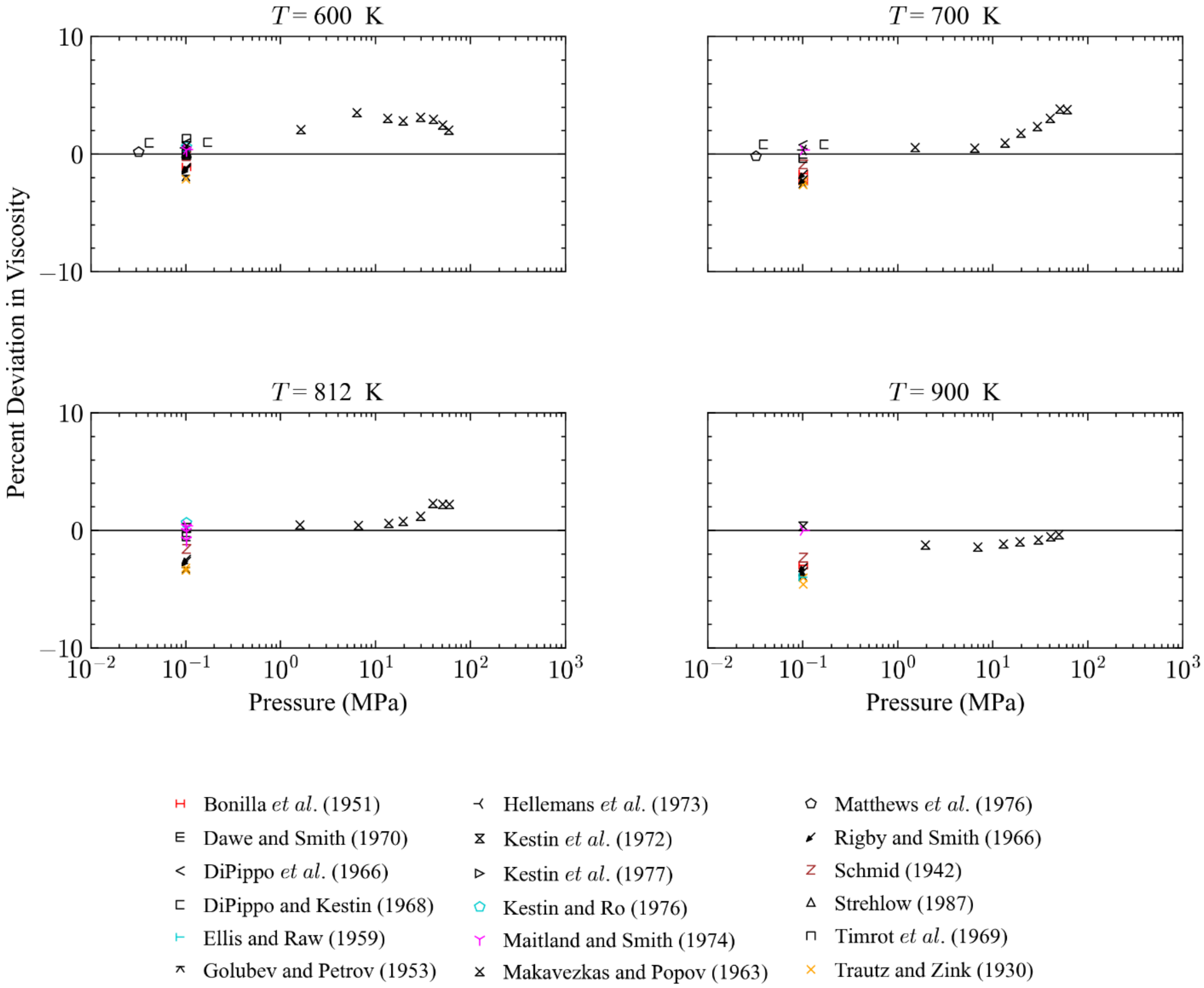

T = 600 K
T = 700 K
T = 812 K
T = 900 K
Percent Deviation in Viscosity
Pressure (MPa)
10
0
−10
10^−2
10^−1
10^0
10^1
10^2
10^3
Bonilla et al. (1951)
Dawe and Smith (1970)
DiPippo et al. (1966)
DiPippo and Kestin (1968)
Ellis and Raw (1959)
Golubev and Petrov (1953)
Hellemans et al. (1973)
Kestin et al. (1972)
Kestin et al. (1977)
Kestin and Ro (1976)
Maitland and Smith (1974)
Makavezkas and Popov (1963)
Matthews et al. (1976)
Rigby and Smith (1966)
Schmid (1942)
Strehlow (1987)
Timrot et al. (1969)
Trautz and Zink (1930)

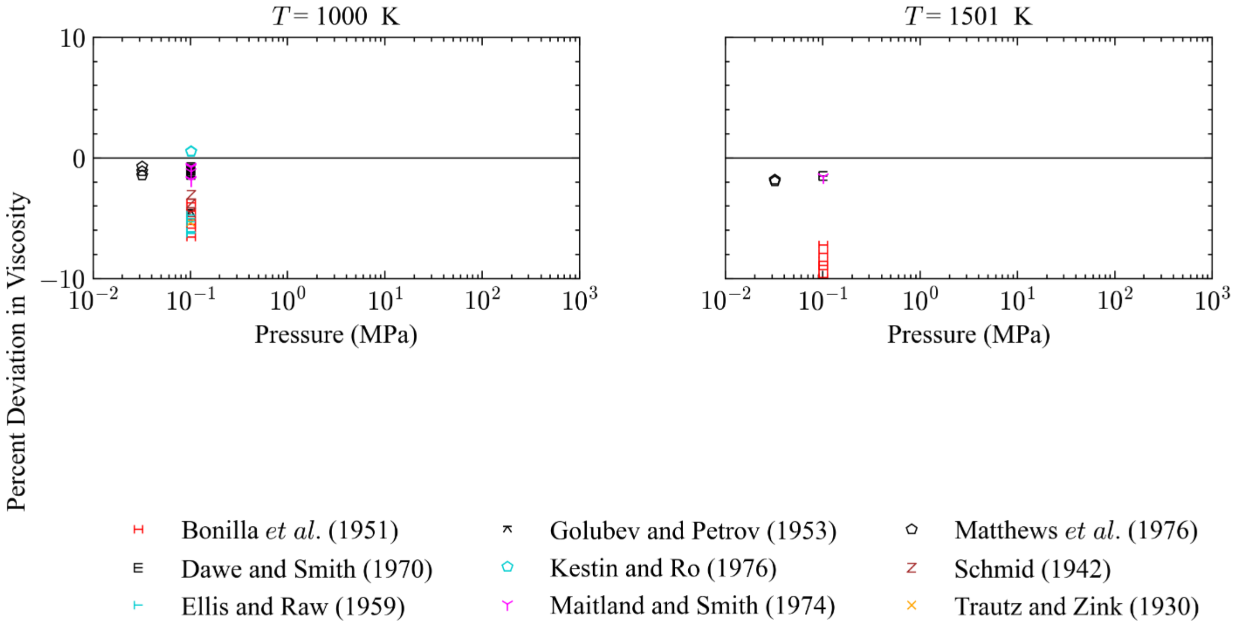

T = 1000 K
T = 1501 K
10
0
−10
10^{-2}
10^{-1}
10^{0}
10^{1}
10^{2}
10^{3}
Pressure (MPa)
Percent Deviation in Viscosity
Bonilla *et al.* (1951)
Dawe and Smith (1970)
Ellis and Raw (1959)
Golubev and Petrov (1953)
Kestin and Ro (1976)
Maitland and Smith (1974)
Matthews *et al.* (1976)
Schmid (1942)
Trautz and Zink (1930)
